# The Wound-Healing Effect of a Novel Fibroblasts-Impregnated Hydroxyethylcellulose Gel in a Rat Full-Thickness Burn Model: A Preclinical Study

**DOI:** 10.3390/biomedicines12102215

**Published:** 2024-09-28

**Authors:** Yury A. Novosad, Anton S. Shabunin, Natella I. Enukashvily, Olga V. Supilnikova, Anastasia I. Konkina, Natalia Yu. Semenova, Gleb S. Yatsemirsky, Evgenii V. Zinoviev, Kristina N. Rodionova, Kirill L. Kryshen, Antonina Yu. Borodina, Alexander Yu. Makarov, Andrey M. Fedyuk, Alexander D. Nilov, Elena V. Chikulaeva, Lidiya S. Konkova, Irina S. Chustrak, Veronika V. Traxova, Platon A. Safonov, Sergey V. Vissarionov, Egor M. Prikhodko, Yury V. Yurkevich

**Affiliations:** 1 Professor G.I. Gaivoronsky Laboratory of Experimental Traumatology and Orthopedics with Vivarium, H. Turner National Medical Research Center for Children’s Orthopedics and Trauma Surgery, 196603 St. Petersburg, Russia; 2 Institute of Biomedical Systems and Biotechnologies, Peter the Great St. Petersburg Polytechnic University, 195251 St. Petersburg, Russia; 3 Cell Technology Center Pokrovsky, 199066 St. Petersburg, Russia; 4 Cell Technologies Lab., North-Western State Medical University named after I.I. Mechnikov, 191015 St. Peterburg, Russia; 5 Lab of the Non-Coding DNA Study, Institute of Cytology, 194064 St. Peterburg, Russia; 6 Research Department of Pathomorphology, Center for Preclinical and Translational Research, Federal State Budgetary Institution «Almazov National Medical Research Centre», Ministry of Health of Russia, 199034 St. Petersburg, Russia; 7 Saint-Petersburg I. I. Dzhanelidze Research Institute of Emergency Medicine, 192242 St. Petersburg, Russia; 8 “Home of Pharmacy” Center, Leningrad Region, 188663 Kuzmolovsky, Russia; 9 H. Turner National Medical Research Center for Children’s Orthopedics and Trauma Surgery, 196603 St. Petersburg, Russia; 10 Institute of Medicine, St. Petersburg State University, 199034 St. Petersburg, Russia

**Keywords:** burn wound, deep partial-thickness burn, full-thickness burn, fibroblasts, hydrogels, regeneration, wound dressing, regenerative medicine

## Abstract

**Background/Objectives:** The objective of this study was to assess the efficacy of a cell-containing wound dressing based on fibroblasts in hydroxyethylcellulose (HEC) gel for the local treatment of deep partial-thickness and/or full-thickness skin burns in an animal model. **Methods:** The rats (male Wistar, n = 100) were subjected to a full-thickness thermal burn (16 cm^2^). Radical necrectomy was performed one day after the burn. Three days later, the rats were randomly assigned to one of four groups: group 1 (no treatment), group 2 (chloramphenicol and methyluracil ointment, a routine clinical treatment), group 3 (a gel without cells, mock treatment), and group 4 (a dermal fibroblast-impregnated HEC gel). The treatment lasted for five days. The wound-healing process was evaluated by planimetric, cytologic, histologic, and immunohistochemical methods. **Results:** The differences in the rate of wound healing and the characteristics of wound cytology were identified. In the group 4, a regenerative type of cytogram was revealed, characterized by a significantly increased number of fibroblastic cells in comparison to samples from non-treated and mock-treated animals. Biopsy samples of burn wounds from animals in the group 4l demonstrated the presence of mature granulation tissue and a large number of microvessels. The repair process was stimulated, as evidenced by the increased thickness of newly formed granulation tissue and epidermis in the wound zone, elevated cellularity, and enhanced re-epithelialization activity. The number of Ki-67-positive proliferating cells was significantly higher in group 4 than in the control groups). A small number of non-proliferating donor fibroblasts was observed in the wound area 3 days after the end of treatment. **Conclusions:** The cell product is an effective agent for promoting wound healing during the regenerative phase. The experiments demonstrated that a gel populated by dermal fibroblasts can stimulate reparative regeneration processes in deep partial- and full-thickness burn wounds.

## 1. Introduction

The issue of providing specialized medical care to victims with partial- and full-thickness burns remains a significant concern. Fire-related burns alone account for over 300,000 deaths per year, with more deaths from scalds, electricity, chemical burns, and other forms of burns [1]. The distribution of victims by age is not uniform: while industrial burn injuries are mostly suffered by males aged from 21 to 30 years, domestic skin burns occur in children and the elderly. Moreover, the frequency of burns in children is four times higher than in adults: burn injuries account for about 25% of all hospitalizations of children [2]. 

The loss of progenitor cells, which are essential for epidermis and dermis regeneration, is a defining characteristic of burn wounds. The use of cell therapy in the treatment of severe burns has the potential to enhance wound healing, facilitate the replacement of damaged tissue, and promote regeneration of the skin [3]. One of the most promising regenerative technologies in the treatment of skin defects is the use of cultured allogeneic connective tissue cells, fibroblasts. These cells stimulate the restoration of epidermal and dermal skin components by synthesizing extracellular matrix, growth factors, and stimulating the proliferation of their own epithelium [4]. 

The viability and survival of donor cells depends on an optimal environment that not only ensures the viability of cells but also facilitates the adaptation of a cell-based medicinal product to the topography of the wound surface [5]. 

Hydroxyethyl cellulose (HEC), a non-ionic inert water-soluble derivative of cellulose ethers, has commonly used to thicken, stabilize, and emulsify in the cosmetic and pharmaceutical industries [6]. HEC is widely used in tissue engineering and in the pharmaceutical industry due its extremely low toxicity and physical properties [7,8,9]. The chemical structure of HEC matches that of glycosaminoglycans in the dermis [10]. The viscosity of HEC gels is sufficient to immobilize fibroblasts in the wound bed, rendering them an appropriate choice for application onto a wound with minimal pain.

The outcomes of successful studies on the application of cell technologies in the treatment of partial- and full-thickness burns provide a foundation for further research in this field [3,11,12,13]. Nevertheless, the Russian Federation currently lacks cell therapy preparations, including those based on the fibroblasts, that have been granted authorization by the national regulatory authority for use in medical practice. 

The objective of this study was to evaluate the efficacy of a wound dressing comprising fibroblasts in HEC gel for the local treatment of deep partial- and full-thickness skin burns in an animal model. The study demonstrated the wound-healing effect on a model of deep burns. The experiments on male rats demonstrated that a gel seeded with dermal fibroblasts can activate reparative regeneration processes in deep partial- and full-thickness burn wounds, as evidenced by the observed outcomes.

## 2. Materials and Methods

### 2.1. Bioethics

All procedures were conducted in accordance with the National Standard 33215-2014 (Guidelines for the Accommodation and Care of Animals) [14] and the recommendations of the European Commission on the Euthanasia of Experimental Animals. All animal experiments conducted in this study were approved by the relevant authorities at the H. Turner National Medical Research Center for Children’s Orthopedics and Trauma Surgery.

### 2.2. Animals

The selection of an appropriate test system (Wistar rats) and the implementation of the “homologous drug product” model (e.g., rat but not human cells applied to a rat, even if a human cell-based product is under study) approach were based on the requirements for the advancement of biomedical cell products studies at the stage of preclinical testing, as recommended by the national regulator [15]. Wistar rats (*n* = 100, 400–450 g) aged 5–6 months, free of disease and lesions, were obtained from a breeding population at the kennels “Rappolovo.” (St. Petersburg, Russia). All animals were quarantined for 14 days and maintained in separate cages under standard conditions. The animals were fed a rough, succulent, and concentrated pelleted feed, with free access to feed and water. 

**Figure 1:**
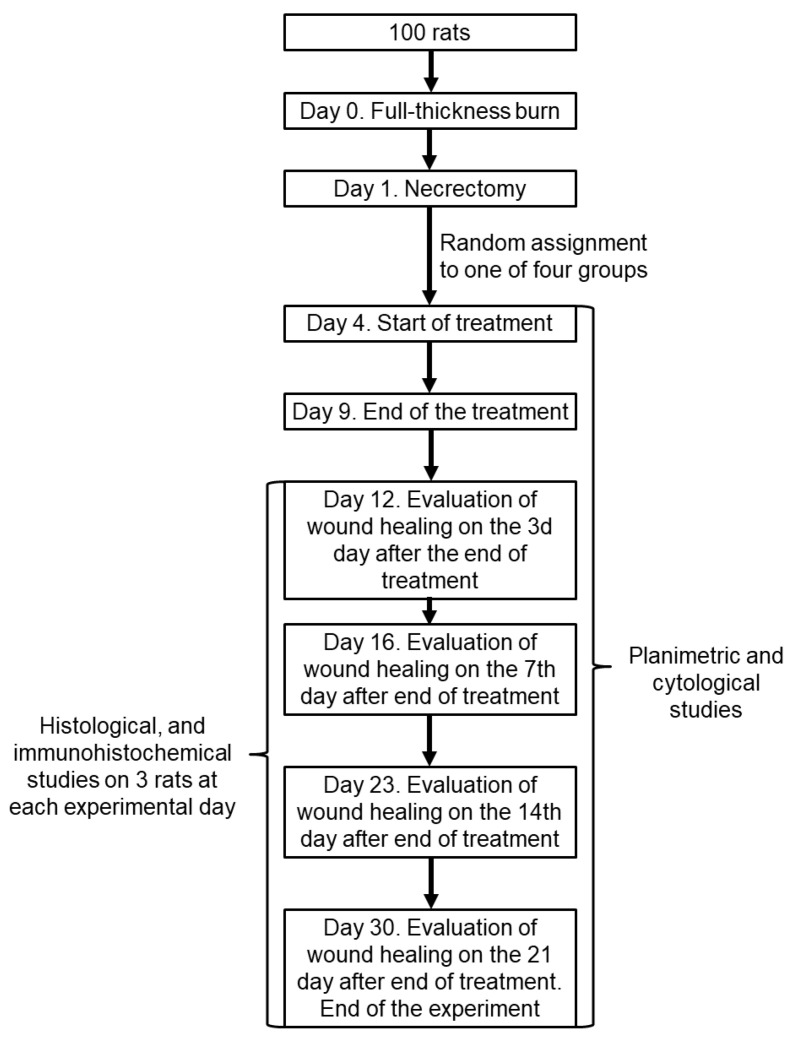
The timeline of the experiment.

The animals were divided into four groups of 25 animals each. Rats of each group were anesthetized and subjected to full-thickness thermal burn, followed by necrectomy in 24 h (Figure 1). The methods employed for the assessment of the wound regeneration dynamics included visual evaluation of the wound surface, planimetric study of wound surfaces, cytologic analysis of wound prints, and histomorphologic and immunohistochemical study of wound biopsy specimens at different periods of observation. The information about animal groups is given in Table 1.

The inclusion criteria were met by observing the homogeneity of the groups in terms of body weight, age, number of individuals, adherence to the rules of selection and maintenance of animals, and the use of the principle and standards of cell preparation. Additionally, the absence of necrotic tissues on the wounds before the application of the preparations was confirmed.

### 2.3. The Study Schedule

The study employed the following checkpoints: prior to the application of the preparations (3 days following the necrectomy), as well as 1, 3, 7, 14, and 21 days following the removal of the therapeutic dressings, which corresponded to 4, 9, 11, 15, 22, and 29 days following the burn (Figure 1). Histomorphological studies (light microscopy, quantitative histomorphology, and immunohistochemistry) were conducted on 12 animals from each group at 3, 7, 14, and 21 days post treatment (Figure 1). 

### 2.4. Medical Intervention

The anesthesia of laboratory animals was conducted using a combined technique, which involved both injection and inhalation. Prior to the administration of anesthesia, the animals were premedicated with atropine sulfate (Dalchimpharm, Khabarovsk, Russia) at a dosage of 0.05 mg per 100 g of animal weight. The induction phase was initiated by placing the animal in a chamber containing sevoflurane vapor (Abbott Laboratories, Sittingbourne, UK) at a concentration of 8%. Subsequently, anesthesia was maintained by infusing 3% sevoflurane into the mask.

Prior to the intervention, the area to be operated on was prepared by depilating the back and marking a 4 × 4 cm square. Subsequently, a heat-resistant silicone mat measuring 3–5 mm in thickness and featuring a cut window aligned with the pre-marked boundaries was positioned on the surgical field. Subsequently, an eight-layer gauze, previously moistened with a 0.9% sodium chloride solution, was positioned on the newly exposed skin area. Subsequently, a heating element with a temperature of 130–135 °C was applied to the skin for a period of 10 s. The area affected by the deep thermal burn was 16 cm^2^. The position and the depth of the burns are shown in Supplementary Figure S1. To calculate the relative area of the burn, the total surface area of the rat body was determined using Meeh’s formula—the surface area of the animal body (cm^2^) can be calculated using the following equation: S = k × W^0.66^, where S is the surface area of the animal body, W is the mass of the animal body, and k is the coefficient, which is 9.13 for the rat [16]. The calculated burn area in relation to the total body surface area of the rat was 3.0–3.2%. The absence of lethal outcomes at a burn area of this size permitted the study to be conducted within the planned time frame, which was a notable advantage.

Twenty-four hours after the burn, the lesion was excised under inhalation anesthesia (3% sevoflurane) in aseptic conditions. This procedure, termed radical necrectomy, aligns with the clinical practice of burn treatment and involves the removal of damaged tissues in the lesion area up to the native fascia [17,18]. Subsequently, muscle sutures were applied at a distance of 1 cm to secure the wound edges and prevent premature closure due to primary tension caused by the rodents’ skin and subcutaneous fatty tissue structure [19]. Further, sterile gauze dressings moistened with a 0.9% sodium chloride solution were fixed over the wound at 12 h intervals per day to maintain the wound moisture until the commencement of treatment. Three days following necrectomy, the animals were randomly distributed into one of four groups, with 25 animals in each group. Control group 1 consisted of untreated animals (Table 1). Animals in control group 2 (Table 1) were treated with chloramphenicol and methyluracil ointment (Laevomecolum, a standard clinical treatment). The wound dressings were changed daily. In control group 3 (Table 1), a HEC gel devoid of cells was applied to the wound surface. The animals in the fourth group (the main group, Table 1) were treated with rat fibroblasts embedded in HEC gel, which was applied to the wound surfaces. On the first day of treatment, the preparations were applied to the burn wounds. Subsequently, all animals, including those in the control group, underwent wound fixation with an indifferent dressing following the application of the aforementioned preparations. The dressing was maintained in a moistened state by covering it with gauze soaked in 0.9% sodium chloride and secured in place with a specialized fixation device. In the control groups and the main group, the wound dressings were not removed during the treatment period. The treatment period for all groups was five days, after which the wound dressings were removed.

### 2.5. Isolation and Expansion of Rat Fibroblasts 

Dermal tissue samples were taken from newborn rats of the same breeding line (Wistar). The dermal tissue was cut into small pieces with scissors and subjected to enzymatic digestion in a solution of 0.1% collagenase types I and IV (Worthington, Lakewood, NJ, USA) in phosphate saline buffer (PBS) for one hour at 37 °C on a stirring platform and then centrifuged. Subsequently, the pellet was treated with a 0.025% trypsin/EDTA solution combined with a 0.1% solution of collagenase types I and IV (Worthington, Lakewood, NJ, USA) in PBS (Biolot, St. Petersburg, Russia), followed by centrifugation. Then, the pellet was re-centrifuged and resuspended in low-glucose DMEM medium (Thermofisher, Gibco, Waltham, MA, USA) supplemented with 10% fetal bovine serum (Thermofisher, Gibco, USA) and penicillin/streptomycin (100 units/100 μg, Thermofisher, Gibco, Waltham, MA, USA). Cells were passaged every three days. The obtained cell culture was subjected to a series of tests to ensure the stable normal karyotype, morphology, and expression of marker mRNAs, as well as the absence of infectious antigens and bacterial and viral contaminants. Following this, the cell culture was frozen. A week prior to the embedding cells into HEC gel, the standard procedure for thawing the fibroblast cultures was carried out, followed by in vitro expansion. Three days prior to the in vivo experiments and gel preparation, the medium was replaced with serum-free StemPro™ MSC SFM medium (Thermofisher, Gibco, Waltham, MA, USA).

The concentration of fibroblasts in the preparation was 500,000 cells/mL of sterile 2% aqueous buffered isotonic HEC gel. The cells and gel concentrations were identical to those planned in the advanced therapy medicinal product. 

### 2.6. HEC Gel Preparation and Seeding with Fibroblasts

Gel carrier is preferred in topical drug delivery system for the direct treatment of a cutaneous disorder [20]. The hydrogel base was prepared using HEC as a gelling agent. This carrier is known for its good rheological properties and is widely used for topical gels (e.g., Diclofenac). To prepare the HEC gel, HEC powder (pharmaceutical grade, Natrosol™, Ashland, Wilmington, DE, USA) was dispersed in phosphate-buffered saline (PBS) and stirred at 37 °C to obtain a uniform dispersion, autoclaved, and stored at +4 °C. To add cells, the gel was reheated to 37 °C and mixed with cells resuspended in a small volume (1/10 of the gel carrier) of 0.9% sodium chloride solution. The details of the gel viscosity vs. velocity (dynamic viscosity) evaluation, which confirmed a non-Newtonian pseudoplastic flow behavior of both the empty HEC gel and the gel impregnated with fibroblasts, are given in the Supplementary File (Supplementary Figure S2, Supplementary Table S1). The viscosity at low speed (0.3) for 2% HEC gel was 223,000 centipoise (cP) or 223 Pa*s) at room temperature (RT) and 170,000 cP (170 Pa*s) at 37 °C. The values obtained corresponded to those published before [21]. The viscosity of HEC gel containing fibroblasts was lower: 77,000 cP (77 Pa*s) at RT and 47,000 cP (47 Pa*s) at 37 °C. These values were chosen experimentally previously [11] to provide (1) easy application (at RT) to the burned skin with minimal pain, and (2) a convenient medium for cells and wound dressing at 37 °C.

### 2.7. Evaluation of Fibroblasts Viability in the Gel

Cell viability was assessed by flow cytometry. Cells were washed out of the water-soluble HEC gel with PBS after 24 and 48 h of storage, stained with propidium iodide, and their viability was assessed by flow cytometry using a Navios flow cytometer (Beckman Coulter, Brea, CA, USA). Viability was estimated by forward (FS) and side (SSC) scattering along with staining with propidium iodide. 

The cells washed from the HEC gel after 48 h of storage were seeded into cell culture flasks and grown in the cell culture medium described above. Cell density and morphology were visually evaluated after 24 and 120 h of cell culture expansion.

### 2.8. MRI Scanning of Fibroblasts Labeled with Iron Oxide Nanoparticles

Fibroblasts were labeled with uncoated iron oxide nanoparticles as we described earlier [22]. The viability of cells before mixing with HEC gel and after it but before injection was controlled with Trypan blue staining followed by counting the cells in a cell counting chamber. The viability of all samples was in the range of 80–92%. The HEC and HEC + fibroblasts gels were prepared as described above. Empty HEC carrier and HEC + scaffold gel were injected subcutaneously. This way of application was chosen for better and longer immobilization of the gels in the body of experimental animals, taking into account the proven non-toxicity of HEC gels when administered subcutaneously [23]. The animals were subjected to magnetic resonance imaging (MRI) scanning of the whole body the next day and on day 6 after injections using Philips Achieva 1.5T MRI scanner (Philips, Amsterdam, The Netherlands). The following MRI sequences were used:

T2 TSE sag (FOV 270*249, TE 95 ms, TR 3000 ms, Matrix 416*352, Slices 13, ST 3.4 mm, Voxel 0.65*0.71);

T1 TSE sag (FOV 190*190, TE 10 ms, TR 550 ms, Matrix 316*252, Slices 21, ST 3 mm, Voxel 0.6*0.75);

T1 TSE ax (FOV 190*190, TE 10 ms, TR 550 ms, Matrix 292*233, Slices 33, ST 4.5 mm, Voxel 0.65*0.81);

T2 TSE ax (FOV 190*190, TE 80 ms, TR 3000 ms, Matrix 344*291, Slices 33, ST 4.5 mm, Voxel 0.55*0.65);

PD TSE ax (FOV 170*170, TE 30 ms, TR 3884 ms, Matrix 284*235, Slices 33, ST 5 mm, Voxel 0.6*0.72);

T2 FFE cor (FOV 170*170, TE 9.2 ms, TR 247 ms, Matrix 172*212, Slices 13, ST 3.5 mm, Voxel 1*0.8);

T2 FFE ax (FOV 149*149, TE 9.2 ms, TR 547 ms, Matrix 148*186, Slices 29, ST 5 mm, Voxel 1*0.8).

### 2.9. Evaluation of the Main Outcome of the Study

The key criteria of local changes in the wound area were visual assessments of the wound surface condition, wound surface planimetry, cytological picture of wound prints, histomorphological examinations of burn wound biopsy in the phases of inflammation and regeneration, and immunohistochemical analyses of proliferative activity of epidermis and dermis cells stained with an antibody against Ki-67 protein. Statistically significant differences, indicating qualitative and quantitative differences in wound healing, were a characteristic of the specific efficacy of the medicinal product under study.

### 2.10. Additional Study Outcomes Evaluation

Additional expected outcomes of the study are related to the wound-healing effect of Chloramphenicol and methyluracil ointment (Laevomecolum).

### 2.11. Visual (Macroscopic) Assessment of the Wound Surface

A macroscopic assessment of the wound-healing process was conducted to evaluate the severity and duration of inflammatory signs in the wound area. These signs included edema, hyperemia, infiltration of paravulnar tissues, the amount and composition of purulent discharge, as well as the time of scab rejection, granulation appearance, and complete healing [24]. 

### 2.12. Planimetric Study of Experimental Wounds

The measurement of the wound area was carried out according to the method developed by L.N. Popova [25]. The relative healing indices were calculated according to the percentage of the wound surface area reduction relative to the primary area and previous values in the studied groups, as well as the burn healing index.

### 2.13. Cytological Study of the Wound

Cytologic study of burn wounds was performed by the method of “wound prints” according to the method of M.P. Pokrovskaya and M.S. Makarov modified by D.M. Shteinberg [25,26,27]. The wound prints were taken from at least 2–3 areas of the wound surface from the center to the periphery. The preparations were dried, fixed in Nikiforov fixative (ethanol-ether, 1:1), and stained according to the Romanowsky–Giemsa method (azur-eosin diluted with distilled water 1:10). After staining, the wound prints were examined under a light microscope “MIKMED-6 LOMO” (LOMO, Saint-Petersburg, Russia) equipped with a Nikon Digital Sight 1000 color camera. At least 5 fields of view (total magnification 1600) in different parts of the preparation were examined. The following parameters were utilized for evaluation: total cell count, a quantitative determination of the ratio of polymorphonuclear leukocytes (including the percentage of destructive forms), lymphocytes, monocytes, macrophages, polyblasts, and fibroblasts within the field of view. The general conclusion on cytograms was based on the definition of the cytogram type according to M.F. Kamaev, modified by O.S. Sergel and Z.G. Goncharova (1990) [28]. The following classification of cytograms was used: necrotic, degenerative-inflammatory, inflammatory, inflammatory-regenerative, and regenerative (with an additional regenerative–inflammatory subclass). The cytograms classification correspond to the sequence of events that occur during the initial phase of the wound-healing process (inflammation), and the subsequent phase, regeneration.

### 2.14. Histological Examination

To prepare histologic specimens, 0.5 × 1 cm fragments of animal skin were excised, including the central region of the burn wound and its periphery with adjacent skin. Samples were fixed in 10% neutral buffered formalin, dehydrated, embedded in paraffin using an automated tissue processor, and sectioned at 3 μm. These sections were then deparaffinized, dehydrated, and stained with hematoxylin and eosin, in accordance with the manufacturer’s recommendations (Biovitrum, Moscow, Russia). The sections were examined under transmitted light (×100, ×200, and ×400) and photographed using an AxioStar microscope (Carl Zeiss, Oberkochen, Germany) and LOMO BLM (LOMO, Saint-Petersburg, Russia). Images were acquired using the MS-cam camera (LOMO, Saint-Petersburg, Russia). ION-labelled cells were revealed with Perls Prussian Blue staining. 

### 2.15. Quantitative Histomorphometry and Immunohistochemical Evaluation of Cell Proliferatiion

A histomorphometric evaluation was conducted on preparations stained with hematoxylin and eosin. The presence of immature granulation tissue and dense granulation tissue/scars was taken as an indication of the extent of regeneration. These characteristics were evaluated semi-quantitatively on a scale from 0 to 3, with 0 indicating the absence of the characteristic, 1 indicating a weak presence, 2 indicating a moderate presence, and 3 indicating a strong presence. To evaluate the epithelium thickness, depth of inflammatory infiltration, cell count in the lesion zone, and granulocyte number in the inflammatory infiltrate, scanned images were analyzed using the Pannoramic Viewer Version 1.15.4 and ORBIT IMAGE ANALYSIS Version 3.64 software.

For immunohistochemical studies, sections (2–3 µm) were deparaffinized. Antigen retrieval was conducted in a water bath (ELMI) at 98 °C and a pH of 9.0 for a period of 25 min. To prevent non-specific binding, 1% bovine serum albumin (BSA) was applied for 30 min in a humidified chamber at room temperature. Samples were then incubated with a polyclonal antibody (AB) against Ki-67 (1:500) raised in rabbit (Cell Marque, Rocklin, CA, USA) for 18 h at 4 °C in a humidified chamber. The Emerald diluent (Cell Marque, Rocklin, CA, USA) was used for the dilution. An alkaline phosphatase-labeled goat anti-rabbit secondary AB (Roche, Basel, Switzerland) was used for visualization. Nuclei were then counterstained with Mayer’s hematoxylin (ErgoProduction, Saint-Petersburg, Russia). Sections were dehydrated, cleared in xylene, and mounted in synthetic mounting medium Vitrogel (ErgoProduction, Saint-Petersburg, Russia).

The quantification of the staining in the epidermis and dermis zones of a 1 mm^2^ burn wound fragment was performed using scanned images with the Pannoramic Viewer Version 1.15.4 software. The number of Ki-67+ cells was counted, as well as the total number of cells. Based on the average values, the proliferation index was calculated using the following formula: 

I_Ki-67_ = N_Ki-67_/N_n_ × 100%, where I_Ki-67_—proliferation index (%); N_Ki-67_—number of Ki-67-positive cells per 1 mm^2^; N_n_—total count of cells per 1 mm^2^.

### 2.16. Fluorescent In Situ Hybridization (FISH)

Histological sections of the wound area taken from female rats on day 12 after the burn (3 days after the end of treatment) were layered onto superadhesive Snowcoat X-TRA slides (Leica, Wetzlar, Germany), deparaffinized with xylene, and dehydrated in ethanols. The rat SRY gene fragment was Cy3-labelled by PCR using the primers described earlier [29] and used as a male-specific probe. The Hybrizol VII (MP Biomedicals, Irvine, CA, USA) was mixed with the probe and applied to the sections. The slides were heated to denature the probe and target DNAs and left for overnight hybridization at 37 °C in a humidified chamber. Slides were then washed in 2× SSC at 41 °C for 10 min, 1× SSC at RT for 10 min, and 0.5× and 0.25× SSC for 5 min each. Slides were then briefly washed in water, air-dried, and mounted in a DAPI-containing antifade mounting medium (Thermofisher, Waltham, MA, USA). Image acquisition was performed using an Olympus FV3000 confocal microscope (Olympus, Tokyo, Japan). To detect DAPI and Cy3 fluorescence, the 405 and 561 nm diode lasers were used for excitation, respectively. The optical sections (0.8 mkm) were taken and stitched to obtain images of areas 1 mm^2^ using MATL (multi-area time lapse) protocol built- in Olympus FV3000 software (https://www.olympus-lifescience.com/en/laser-scanning/fv3000/multi-area-time-lapse-software-module/, accessed on 22 September 2024). The number of Cy3-positive cells was calculated for these five stitched images.

### 2.17. Statistical Analysis

Statistical processing of the data was performed in the software environment of STATISTICA 10 package (Tibco, Round Rock, TX, USA) and using Wolfram Mathematica 11.0 software (Wolfram, Champaign, IL, USA). Graphical editors of STATISTICA 10 and Microsoft Office packages were used to visualize the results of statistical analysis.

The distribution of quantitative indicators was evaluated for compliance with the theoretical Gaussian law (normal distribution) using the Shapiro–Wilk test of normality. In the description of quantitative variables, medians (Me) were employed as a measure of central tendency, while lower (Q1) and upper (Q3) quartiles were utilized as a measure of variability. The Kruskal–Wallis rank test of variance was conducted, and in the event of a statistically significant result, a pairwise comparison of groups was performed using the Mann–Whitney test. When evaluating qualitative indicators, the values of the *χ*2 criterion (chi-square) and Fisher’s test were calculated. The observed differences were considered statistically significant at a two-sided significance level of *p* ≤ 0.05.

The statistical processing of the primary data obtained through the use of morphometric and immunohistochemical methods was conducted using the software program StatTech v. 3.1.10 (StatTech LLC, Moscow, Russia).

## 3. Results

### 3.1. Cells Viability in the Gel

**Figure 2:**
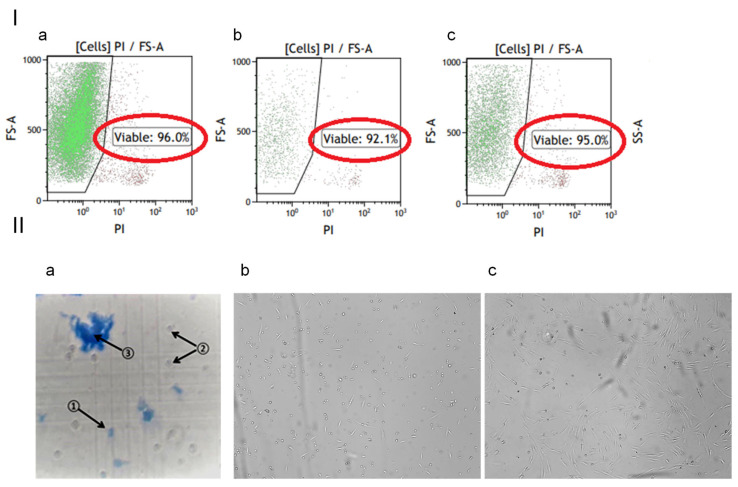
Viability of cells in the gel. (**I**) Flow cytometry of non-permeabilized cells stained with propidium iodide (PI): (**a**) fibroblasts before loading into the HEC gel, (**b**) washed out of the gel at 24 h, and (**c**) at 48 h after loading into the HEC gel. The viable cells were not stained with PI and are therefore on the left side (black polygonal line) of the plots. (**II**) (**a**) Cells washed from the gel 48 h after loading were seeded and observed 24 h (**b**) and 120 h (**c**) after seeding. The image in IIa was taken from the cell counting chamber, cells were stained with trypan blue, (1)—scaffold fragments, (2)—live unstained cells, (3)—dead cells stained with the dye. Magnification 50×.

The viability of cells washed out from the HEC gel scaffold was studied in flow cytometry experiments (Figure 2I(a–c)). The 24 h and 48 h control points of storage at room temperature were chosen because the intended shelf life was 48 h. No significant difference was observed in the viability of cells removed from gel 24 and 48 h after preparing cells-impregnated gel as compared to the same cells before introducing the into HEC scaffold. 

The cells, which were washed out of the gel 48 h after its preparation, were seeded onto cell culture flasks and observed for 5 days without changing the medium (Figure 2II(a–c)). After this period, the cells adhered to the surface, and the morphology of the cells was similar to that of fibroblasts.

### 3.2. Assessment of the Distribution of Cells

**Figure 3:**
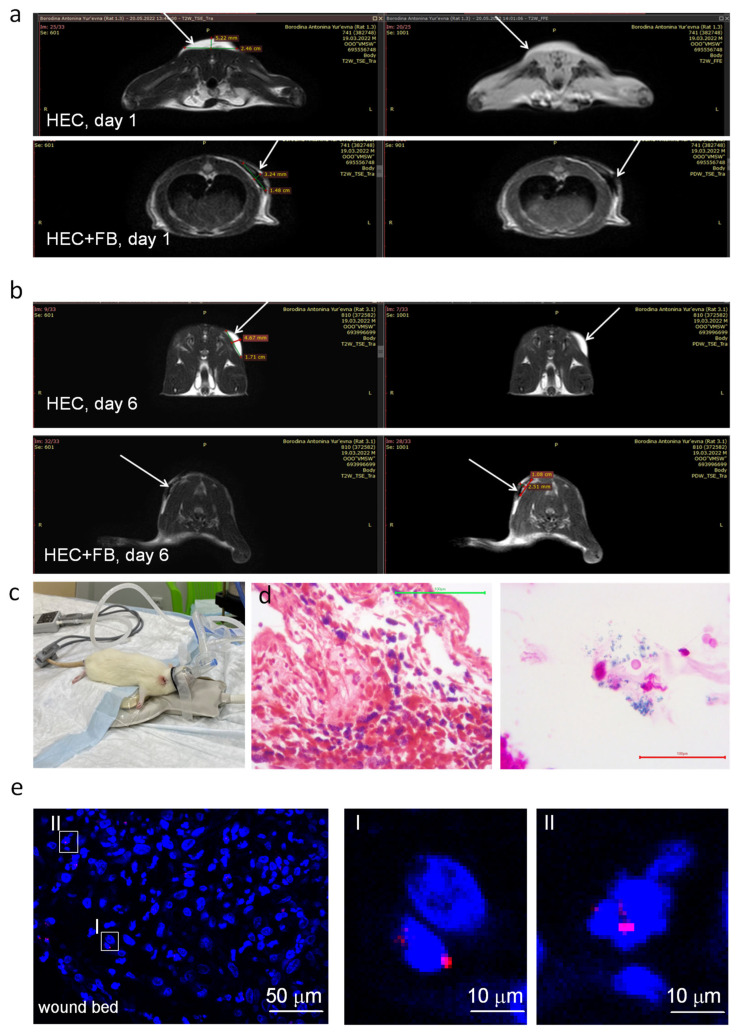
Fibroblasts migration of the HEC gel. (**a**,**b**) Empty HEC gel (top rows in (**a**,**b**)) and fibroblasts (FB) labeled with uncoated iron oxide nanoparticles (bottom rows in (**a**,**b**)) and embedded in the HEC gel were injected subcutaneously (white arrows) into rats. The animals were MRI scanned (**c**) the next day (**a**) and 6 days (**b**) after injection. After scanning, the site of injection was excised and examined histologically using Perls Prussian Blue staining (**d**), which stains the iron oxide nanoparticles blue. The images in (**d**) were taken the next day (left image) and 6 days (right image) after injection. The presence of allogeneic fibroblasts in the burn wound was checked on day 12 of the experiment (3 days after the end of treatment) by fluorescence in situ hybridization (FISH) (**e**). Male fibroblasts in HEC gel were applied to the burn wound. On day 12, the wound area was excised, and paraffin sections were used for FISH with Y-chromosome probe (red). A confocal section (0.8 mkm) is shown. Nuclei were counterstained with DAPI. I, II—areas at a larger magnification. Scale bars are shown in the images.

The distribution of allogeneic fibroblasts in the wound and their ability to exit the gel was assessed by FISH, histologic examination, and MRI (Figure 3). 

The ability of fibroblasts to migrate out of the HEC gel was evaluated in experiments with subcutaneous injection of empty and fibroblast seeded HEC gels. The gels were injected but not applied to the skin surface for a better evaluation of cell distribution—the gel remained under the skin for a longer period of time, providing better conditions for the registration of cell migration. The biological distribution of fibroblasts labeled with iron oxide nanoparticles (IONs) was evaluated by MRI (Figure 3a–c). The HEC scaffold was saturated with water and therefore appears white in the MRI images (Figure 3a,b, top rows). Proton-depleted IONs acted as contrast agents in MRI, causing a decrease in signal intensity. Therefore, an area where labeled cells are clustered is black in the MRI images. Both the empty HEC gel (white in the MRI images) and the gel impregnated with ION-labeled fibroblasts were detected at the site of their injection both the next day and 6 days after injection (Figure 3a,b, bottom rows). The areas of injected substances remained compact without detectable tracks of diffusing labeled cells. The measurements on day 1 and day 6 were made on different animals because the animals were terminated after the scanning for histological examination (Figure 3d). The next day after injection, clusters of cells with IONs were detected at the gel localization site (Figure 3d, left image). On day 6, the number of labeled cells decreased (Figure 3d, right image), although the injection site was still detectable on MRI (Figure 3b). Thus, fibroblasts remained mostly within the gel for 6 days, although some cells left the HEC gel. When the fibroblast-loaded HEC gel was applied to the experimental burn wound (Figure 3e), a small number of fibroblasts (2–8 per scanned image area 1 × 1mm) were detected in the granulation tissue and under the wound bed on day 12 of the experiment, i.e., 3 days after the wound dressing containing the gel was removed. The gel carrier was not detected on the wound surface or below. Thus, despite the degradation and mechanical gel removal, some single cells could leave it. Clusters of fluorescently labelled cells or neoplasms were not observed, suggesting that allogeneic fibroblasts did not proliferate in the wound area. 

### 3.3. Vusual Examination of the Wound Surface

**Figure 4:**
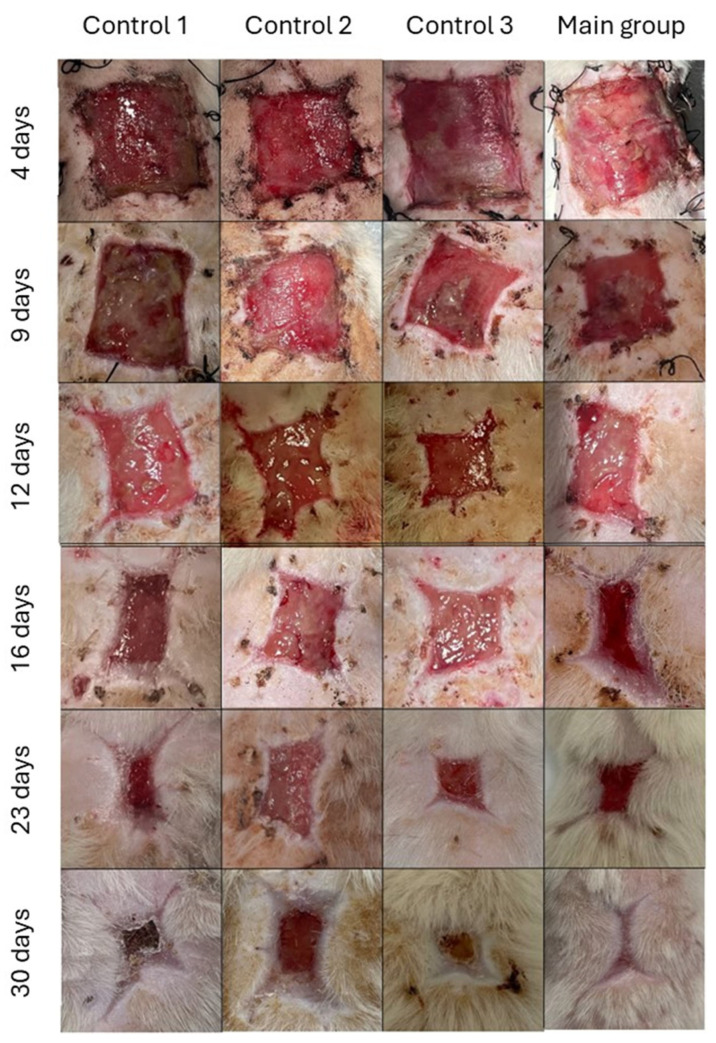
Visual examination of the wound in experimental groups. The timeline corresponds to the timeline in Figure 1: 4 days—start of treatment, 9 days—end of treatment, 12, 16, 23, 30 days—3, 7, 14, 21 days after treatment.

Prior to the commencement of treatment, three days post necrectomy (i.e., 4 days after the start of the experiment), the wounds exhibited a rectangular shape with a bright red bottom (Figure 4, the top row). 

A pronounced local inflammatory reaction was observed, accompanied by a marginal hyperemia extending up to 0.3–0.5 cm. Additionally, there was a noticeable swelling of skin tissues at the border with the wound surface. A thin layer of fibrinous purulent discharge was also observed, which was easily washable. The preparations were applied after thorough cleansing of the burn wounds. One day after the end of the treatment and removal of the therapeutic dressings, the superficial layers showed a crust-like appearance. The burn wound in control group 1 exhibited fibrinous purulent discharge. In addition, areas of suppuration were observed in the groups that received gel application and cell preparation, with the majority of these occurring in the central zone of the wound surface. The least pronounced signs of inflammation and edema were observed in the group treated with chloramphenicol and methyluracil (Laevomecolum) ointment. Only narrow marginal areas of suppuration were observed in the area of fixation sutures. On day 12 (3 days after treatment), the wound surface of all animals was covered with granulations and areas of fibrinous purulent plaque, including those treated with the ointment preparation (Figure 4). After seven days (day 16 of the experiment), the severity of inflammatory changes in the wounds of animals in all groups decreased, and early signs of wound healing were observed, including the sloughing of scab edges and the appearance of areas of marginal epithelization (Figure 4). These changes were most variable in the main group. The least pronounced signs of inflammation and edema were observed in the group treated with Laevomecolum ointment. Only narrow marginal areas of suppuration were observed in the area of the fixation sutures. In control group 1 (without wound treatment), granulations formed unevenly. After 14 days (day 21 of the experiment), the wound surfaces of animals in all groups were covered with granulations under the scab (Figure 4). In the group of animals with applied cell-seeded HEC gel, distinct signs of active scab rejection and marginal epithelization with exposure of young bright pink skin were observed. By the end of 21 days after treatment (day 30 of the experiment), zones of marginal epithelization were observed in all groups of animals. The size of the wound surface significantly decreased. In the main group (treated with cell-seeded HEC gel), scab rejection and complete epidermalization of wounds were observed (Figure 4). In control group 2 (treated with Laevomecolum), the healing process was less advanced. In the central zone, the wound was covered with a dense crust that prevented epithelization. Healing of burn wounds in control groups 1–3 was observed no earlier than 35 days and later.

### 3.4. A Planimetric Assessment of Wound Surface Healing

Immediately prior to the commencement of treatment (four days post burn), the wound areas in all groups exhibited comparable characteristics (Table 2, Figure 4). 

One day after the end of treatment (nine days after the burn), the burn area was reduced, with the greatest reduction observed in animals treated with Laevomecolum ointment. In control group 2, the burn wound area decreased from 13.57 cm^2^ to 8.97 cm^2^, a reduction of 33.9% (Table 2). In untreated control group 1, the wound area decreased by a maximum of 8.2% (from 13.45 cm^2^ to 12.35 cm^2^). The relative difference in burn wound-healing area between the two groups was 25.7% (*p* ≤ 0.05). The application of a HEC gel scaffold without fibroblasts to the burn wound in control group 3 resulted in a reduction of the wound surface area of no more than 10.2% relative to the primary area (from 13.22 cm^2^ to 11.42 cm^2^), with no significant differences from the untreated control group 1. The application of a gel seeded with fibroblasts in the main group resulted in a reduction of the wound surface area of 24.4% (from 13.81 cm^2^ to 10.44 cm^2^). The rate of relative healing of burn wounds in this group compared to the control group treated with Laevomecolum was 14% lower (*p* ≤ 0.05).

By the end of the third day following the end of the treatment period (11 days post burn), the wound areas in control group 2 and in the main group did not differ significantly (Table 2). The median burn area was 6.17 cm^2^ (5.40–6.89 cm^2^) and 6.57 cm^2^ (6.10–7.18 cm^2^), respectively (*p* = 0.14). The decrease in relative burn area compared to the previous observation period, during which wounds in group 2 were treated with Laevomecolum ointment, was 31.2%, while the decrease when fibroblasts in HEC gel were used was 37% (*p* = 0.12). In control groups 1 and 3, the burn wound area was larger (*p* ≤ 0.05) compared to control group 2 and the main group. The burn wound area in control groups 1 and 3 was 9.63 [Q1 9.10; Q3 9.94] cm2 and 8.87 [Q1 8.40; Q3 9.42] cm^2^, respectively, and did not differ significantly from each other. The relative healing rates in these groups were also statistically indistinguishable (22% and 22.3%, *p* = 0.33).

From day 7 after the end of treatment, the application of fibroblasts to the wounds of the experimental animals resulted in a notable acceleration in the rate of wound healing (Table 2). The wound surface area was reduced to 3.94 cm2 (3.55–4.33 cm^2^). The median burn area of wounds treated with Laevomekolum was 4.55 cm^2^ (3.94–5.01 cm^2^) and was 13.4% (*p* ≤ 0.05) higher than that of the fibroblasts-treated wounds. In comparison to the previous stage of observation, the reduction in the wound surface area in the fibroblasts-treated wounds was 40%, whereas in control group 2, the decrease did not exceed 26.2% (*p* = 0.038). The areas of burn wounds in control groups 1 and 3 were 7.12 cm^2^ (Q1 6.27 cm^2^, Q3 7.74 cm^2^) and 6.37 cm^2^ (Q1 5.82 cm^2^, Q3 7.2 cm^2^), respectively, and did not differ significantly. The analysis of the burn wound area and the rate of wound healing in the main group and group 2 revealed a less-pronounced dynamic in the reduction of the wound surface for control group 2. The relative burn wound-healing rate in the fibroblasts-treated group differed by 44% (*p* ≤ 0.05) from that of animals in the non-treated control group and by more than 40% from that of control 3 group.

A comparison of the burn area between the main group and control groups revealed that, 14 days after the end of treatment, the burn area had decreased in size, and the wound-healing process was actively developing (Table 2). By this point, the burn area in the main group had decreased to 1.42 cm^2^ (Q1.19 cm^2^, Q3 1.63 cm^2^). In control group 2, the area of the non-repaired wound surface was significantly larger, at 2.33 [1.66; 2.72] cm^2^ (*p* ≤ 0.05). The relative area of non-repaired wound also differed significantly. In group 3, the area of the non-repaired wound surface was 3.81 [Q1 2.85; Q3 4.33] cm^2^, which was 19.5% (*p* ≤ 0.05) higher than in the fibroblasts-treated group. The greatest burn wound area (4.54 cm^2^, Q1 4.31; Q2 5.41) was observed in control group 1. This was 1.95 times (*p* ≤ 0.05) greater than in control group 2 and 3.2 times (*p* ≤ 0.05) greater than in the main group.

At 21 days after the end of the treatment period, the accelerated wound area reduction observed in the main group was still evident. The area of the burn injury was reduced to 0.25 cm^2^ (Q 1 0.18, Q3 0.47 cm^2^, as shown in Table 2) and comprised no more than 1.8% of the initial burn wound area prior to treatment. In the majority of animals in the main group, the burn wounds exhibited complete closure with epithelialization and scar formation. The rates of burn wound healing in animals in control group 2 were slower than in the other groups. The residual wound area in group 2 was 1.28 cm^2^, which was 8.6% of the original burn area (*p* ≤ 0.05). A similar wound surface area was observed in the animals of the main group by the end of 14 days after the end of treatment (Table 2). The wound-healing effect of hydroxyethylcellulose gel without cells was less pronounced than that of gel with fibroblasts. The residual area of the burn wound in control group 3 was 1.61 cm^2^, which was 12.2% of the initial burn area. However, in control group 1 (without treatment), the burn surface area was the largest, at 2.57 [Q1 2.26; Q4 3.12] cm^2^. In this group, the relative residual area of the non-closed burn surface was 19.1% of the original burn area, which was statistically significantly different from the main group, as well from control groups 2 and 3. 

Thus, according to the planimetric assay, the wound-healing process was the most intense in the group of animals treated with dermal fibroblasts embedded in gel scaffold.

### 3.5. The Cytological Profile of Wound Prints

**Figure 5:**
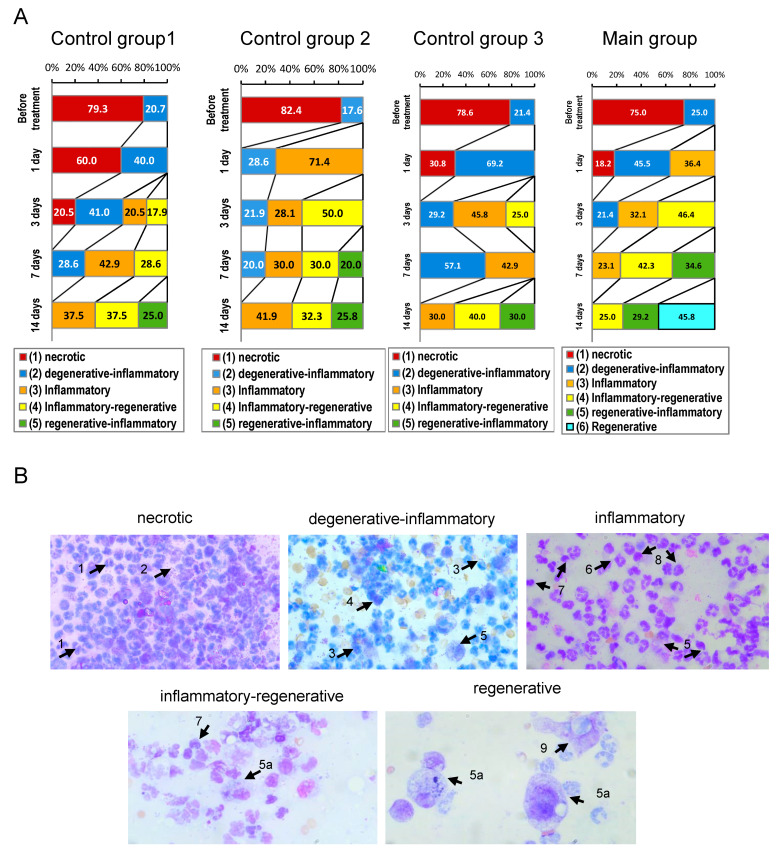
Analysis of cytological profiles (cytograms) frequencies (**A**) in the control and experimental groups. The days after the end of treatment are shown on the left side of each histogram. The cytological profiles were obtained through the analysis of wound prints. Examples of cytograms subtypes are given in (**B**). 1—polymorphonuclear leukocytes with cytological features of cellular destruction, 2—extracellular colonies of microorganisms, 3—polymorphonuclear leukocytes with intracellular and extracellular microorganisms, 4—monocytes, 5—polyblasts, 5a—polyblasts with vacuole, 6—leukocytes without microbial contamination, 7—lymphocytes, 8—macrophages, 9—fibroblasts. Magnification 400×.

Prior to the treatment, the cytological profile of the wound prints in the majority of animals (75–82.4%) was consistent with a purulent-necrotic process. The proportion of cytograms of the degenerative-inflammatory type did not exceed 17.6–25% (Figure 5).

One day after the application of an indifferent dressing to the wound surface (control group 1), the purulent-necrotic type of cytograms was observed in 60% of animals (Figure 5). The remaining cytograms exhibited degenerative characteristics with evidence of an inflammatory response. In control group 2 (treatment with Laevomecolum), there was a significant decrease in degenerative-inflammatory changes in the wound prints. The purulent-necrotic type of cytograms was not observed. The proportion of degenerative-inflammatory cytograms did not exceed 28.6%. The cytograms (71.4%) exhibited inflammatory characteristics, indicating the wound process was in the phase of active inflammation. In control group 3 (mock treatment with no-cell HEC gel), necrotic and degenerative-inflammatory types of cytograms were observed. When fibroblasts were applied to the burn wound surface, the proportion of cytograms with purulent-necrotic characteristics did not exceed 18.2% (Figure 5). A decrease was observed in the proportion of cytograms of degenerative-inflammatory type (from 69.2% to 45.5%), accompanied by an increase in the percentage of cytograms of inflammatory type (up to 36.4%), indicating a positive effect of fibroblasts on the normalization of the inflammatory phase of the wound process. It is also noteworthy that the proportion of inflammatory cytograms in animals treated with Chloramphenicol and methyluracil ointment (Laevomecolum) was 33.6% (*p* ≤ 0.05) higher than in the main group (Figure 5).

At 3 days after the end of treatment, positive dynamics of the inflammatory phase of the wound process with initial signs of regeneration was observed in all groups of animals (Figure 5). In control group 1, the number of purulent-necrotic cytograms decreased (from 60% to 20.5%), and the degenerative-inflammatory type persisted in 41% of animals, indicating a wound process with a weak inflammatory reaction. Additionally, inflammatory cytograms (20.5%) and inflammatory-regenerative cytograms (17.9%) were identified, indicating the active course of the inflammation phase and the transition of the wound process to the regeneration phase. In control group 2 (Laevomecolum-treated), cytograms of purulent-necrotic type were not observed, and destructive-inflammatory forms did not exceed 21.9%. The number of extracellularly located cocco-bacillary bacteria was significantly lower compared to the same period in control group 1, indicating the antimicrobial effect of Chloramphenicol + methyluracil ointment (Laevomecolum). The proportion of cytograms of inflammatory type (28.1%) did not change, and the number of cytograms of inflammatory-regenerative type increased, reaching 50%, reflecting the active development of the period of the regenerative phase of the wound process. In contrast to untreated animals, no cytograms of purulent-necrotic type were detected when empty HEC gel scaffold was applied to the wound surface (Figure 5). The proportion of cytograms of degenerative-inflammatory type remained stable, with only a non-significant decrease from 41% to 29%. At the same time, inflammatory cytograms were observed in 45.8% of cases, representing a 2.2-fold increase (*p* = 0.033) compared to the number of inflammatory cytograms observed in untreated animals. These data confirmed a more active normalization of the inflammatory phase compared to untreated animals. The proportion of inflammatory-regenerative cytograms, indicating the transition of the wound process into the regeneration phase, showed a tendency to increase (from 18% to 25%). In the group where fibroblasts seeded in a gel scaffold were applied to the wound, predominantly inflammatory (32.1%) and inflammatory-regenerative (46.4%) types of cytograms were observed (Figure 5). The absence of necrotic cytograms is noteworthy. The proportion of cytograms of degenerative-inflammatory type did not exceed 21.4%. A comparison of the cytological profile of the wound prints from animals treated with an empty HEC gel showed a significant increase in the proportion of cytograms of the inflammatory-regenerative type (from 25% to 46.4%). These data suggest that dermal fibroblasts may have a stimulating effect on reparative regeneration.

At 7 days after the application of neutral wound dressing (gauze bondages), in control group 1, the cytological profile of wound prints of degenerative-inflammatory type was retained in 28.6% of cases (Figure 5). The transition of the wound-healing process to an inflammatory type was observed (42.9%), and the ratio of cytograms of the inflammatory-regenerative type increased (from 18% to 28.6%), indicating the period of transition from the inflammatory phase of the wound-healing process to the phase of regeneration. In control group 2, the percentage of cytograms of degenerative-inflammatory and inflammatory types was 20% and 30%, respectively. At the same time, the cellular composition of cytograms of the inflammatory-regenerative type decreased from 50% to 30%, indicating a partial transition to the regenerative-inflammatory type, characterized by the accumulation of regenerative undifferentiated and differentiated histiocytic forms (polyblasts, fibroblasts). The application of empty HEC gel to the wound resulted in the observation of further positive dynamics of the inflammatory phase of the wound process, with a transition to the regenerative phase. No cytograms of the degenerative-inflammatory type were observed. In more than half of the cases (57.1%), cytograms of an inflammatory type were observed (Figure 5). The percentage of cytograms of the inflammatory-regenerative type reached 42.9%, which is indicative of the initial period of the regeneration phase. The cytograms of the active phase of regeneration were not determined. In the group of animals treated with fibroblast-seeded HEC gel, an active transition from the inflammatory phase to the regenerative phase of wound healing was observed. The percentage of inflammatory cytograms did not exceed 23.1%. The other cytograms (76.9%) corresponded to different periods of the regeneration phase, including inflammatory-regenerative and regenerative-inflammatory ones (Figure 5). A comparative analysis of cytogram types revealed that the wound process in the cells-treated group was undergoing an active regeneration phase, with the activation of regenerative processes facilitated by the described cell technology.

At 14 days after the removal of the neutral wound dressing in control group 1, cytograms of degenerative-inflammatory type were not detected. The percentage of inflammatory type cytograms was 37.5%, indicating the transition to the regenerative phase of the wound process (Figure 5). Inflammatory-regenerative and regenerative-inflammatory types of cytograms comprised 37.5% and 25% of all cytograms in the group, respectively. The regenerative type of cytograms typical of the second phase of the wound-healing process was not detected. In control group 2, the inflammatory response with transition to the inflammatory-regenerative type was preserved. There were no significant differences in percentage of inflammatory, inflammatory-regenerative, and regenerative-inflammatory types in comparison with control group 1. In control group 3, the dynamics of the transition of the inflammatory phase to the regeneration phase also had no significant differences with the non-treated animals, as well as with Laevomecolum-treated. In wounds of rats with application of fibroblasts in a gel scaffold, there was a more intensive development of the regenerative phase of the wound process in comparison with control groups. The number of cytograms corresponding to successive stages of regeneration was increased. Cytograms of the inflammatory-regenerative type accounted for 25%, and those of the regenerative-inflammatory type—29.2%. The characteristic feature of the regeneration period in this group was the appearance of cytograms of the regenerative type (Figure 5). The percentage of such cytograms reached 45.8%, which indicates accelerated development of the regenerative phase.

### 3.6. The Histological Examination of Biopsy

**Figure 6:**
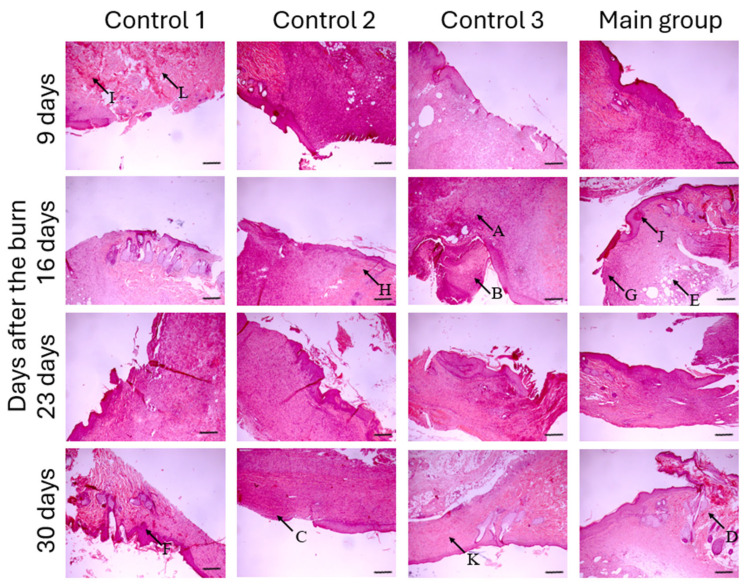
Morphology of the defect area. In control groups 1 (untreated) and 3 (HEC gel without cells) without Laevomecolum application, on days 16–30, more significant purulent inflammation (A) under a large scab (B), a smaller volume of granulation tissue, and less significant epidermalization were observed. On day 30, epidermal defects were observed in the Laevomecolum group (control group 2), and the bottom of the defect was covered with scar tissue (C). In the main group (HEC gel seeded with fibroblasts), there was more active growth of granulation tissue on days 16–30 than in the control groups. On day 30, complete epidermalization was observed with the formation of skin accessory structures (sebaceous glands, sweat glands, and hair follicles) and focal hyperocratosis (D). The timeline is the same as shown in Figure 1. Scale bar—100 μm. Multiple vessels (E), acanthosis (F), leukocyte infiltrations (G), diapedesis hemorrhages (H), fibrinous exudate (I), keratinous cysts (J), sclerosis (K), edematous areas (L).

On the third day following the removal of the wound dressing in control group 1, the bottom of the wound was formed by a narrow zone of granulation tissue with dense diffuse leukocyte infiltration, multiple vessels filled with blood, edema, and hemorrhages (Figure 6). Along the wound edges, limited, discontinuous areas of keratinizing epithelium with hyperkeratosis and focal massive acanthosis were detected. In seven days after the wound dressing removal (day 16 of the experiment, see Figure 1 for the timeline), evidence of marginal epithelialization became apparent in small areas. The transition of granulation tissue to mature tissue was not observed. The stroma fibers were observed to be loosely arranged with an irregular orientation. A considerable number of leukocyte infiltrations, venous hemorrhages, and diapedesis hemorrhages was observed. A defect in the epithelial lining, covered by a scab, persisted 14 days after the removal of the wound dressing (day 23 of the experiment). The epidermis consisted of small, thin areas comprising from one to three rows of cells, alternating with regions exhibiting acanthosis. As the marginal epidermal regeneration developed, granulation tissue with a leukocyte infiltrate became increasingly prominent. In the developing dermis, the presence of a fibrinous exudate, and a number of large focal hemorrhages were observed. At the end of the 21-day period, evidence of delayed tissue regeneration remained evident. The newly formed epidermis was observed to be thin and irregularly arranged, with a notable absence in the central zone. Numerous rounded keratinous cysts were observed in the stroma. Most of the connective tissue fibers were observed to be sclerosed with a coating of fibrinous exudate. The inflammatory infiltration observed in the marginal zone of the defect persisted, while scar tissue formation occurred in the deeper layers of the wound. Edematous areas with mature granulation tissue and inflammatory infiltration were observed in the central region of the defect.

In control group 2, three days after the end of the treatment period, evidence of partial re-epithelialization and the initial stages of dense mature granulation tissue formation were observed in the deeper layers of the marginal zone of the defect. The majority of the stroma consisted of granulation tissue with a mild leukocytic reaction (Figure 6). Moderate edema and sclerosis of the fibers were observed, accompanied by impregnation with fibrinous exudate. On day 16 of the experiment, seven days after the end of the treatment period, the marginal areas showed a depleted epidermis with acanthosis and fibrinous plaque. The process of re-epithelialization was more pronounced compared to control group 1. The stroma was represented by mature granulation tissue with leukocyte infiltration, similar to the inflammatory response observed in the animals of control group 1 (Figure 6). At 14 days, the bottom of the wound was covered by epidermis crawling from the edges with focal expressed hyperkeratosis, in some places it was thinned. The inflammatory infiltration was diffuse but became dense in the vicinity of the wound edges. At the wound bed, there was young fibrous tissue with fibrin overlay, foci of lymphohistiocytic infiltration, and large focal hemorrhages. On day 21 after treatment (day 30 of the experiment), the newly formed epidermis was observed to be thin in some areas. The newly formed epithelium showed a multilayered squamous structure with pronounced hyperkeratosis and moderate papillomatosis. The defect of the epithelial lining was preserved. In the marginal zone of the defect, mature granulation tissue was observed, characterized by abundant mixed-cell infiltration and multiple microcirculatory channels.

In control group 3 (empty HEC scaffold), the formation of limited areas of newly developed multilayered squamous keratinizing epithelium was observed at the edges of the wound defect three days after the conclusion of the treatment (Figure 6). At the wound bed, granulation tissue was observed, displaying evidence of microabscesses, necrotic foci, and edema. Extensive fibrinous deposits with a notable leukocyte infiltrate were observed in close proximity to the edge of the desquamated tissue. Following a 7-day observation period (16 days in total), irregular areas of marginal epithelium remained. The majority of the skin defect area was constituted by granulation tissue with a notable leukocytic infiltration and multiple microcirculatory channels. The upper layers of the stroma were composed primarily of fibrous tissue with a minimal presence of keratinized cysts. The lower layers exhibited a notable presence of granulation and fibrous tissue, accompanied by infiltration of leukocytes and lymphocytes. At the end of the 14-day observation period, the marginal epidermis demonstrated thinning layers of multilayered squamous keratinizing epithelium. The process of re-epithelization was more pronounced in comparison to the previous observation checkpoint (which was seven days after the conclusion of the treatment). A broad subepithelial layer of fibrous tissue with elements of sclerosis was observed. The deeper layers of the wound exhibited the presence of granulation tissue fibers with moderate mixed-cell infiltration and evidence of small focal hemorrhages. Two weeks following the removal of the empty gel scaffold, the epidermis was observed to consist of irregular multilayered squamous epithelium with moderate hyperkeratosis. The defect of the epithelial lining was maintained. The bottom of the defect was constituted by mature granulation tissue with a small number of thick-walled vessels. The presence of inflammatory infiltration was observed in the marginal zone of the defect. No significant differences in the morphology of the wound at the final stage of the study were identified in comparison with the animals in the other control groups.

A histological examination conducted three days after the conclusion of treatment in the group treated with dermal fibroblasts embedded in HEC gel revealed the presence of granulation tissue of varying degrees of maturity. The area of eschar localization exhibited a prevalence of dense inflammatory leukocyte infiltration. Microabscesses and dilated full blood vessels of the microcirculatory channel were observed at the bottom of the wound defect (Figure 6). Partial re-epithelialization was observed along the wound edges. On day 7 of post-treatment observation, the marginal epidermis was observed to display uneven thickness and proliferation of the basal layer. At this checkpoint, the presence of subepidermal mature granulation tissue and the growth of capillary-like vessels were discernible. The total number of cells in the field of view was increased, predominantly due to the presence of fibroblastic cells. No evidence of inflammatory infiltration was observed. On day 14, active marginal epidermalization of the wound defect was evident. Mature granulation tissue with thickened vessel walls and perivascular lymphoid infiltration was found in the subepithelial tissue. Numerous fibroblasts and plasmocytes were seen in the samples. In some areas, layers of fibrous connective tissue with lympho-plasma–macrophage infiltration, fields of newly formed capillary-like vessels, and the overgrowth of fibrous structures were observed. After 21 days, a restored epidermal layer was observed, filling the damaged area from the edges of the wound defect. Subepithelial stromal areas were detected as mature granulation tissue. The formed dermis was free of inflammatory infiltration. Regular fibrous structures with capillary-like vessels prevailed. Fibroblasts predominated among the cells detected (Figure 6).

**Figure 7:**
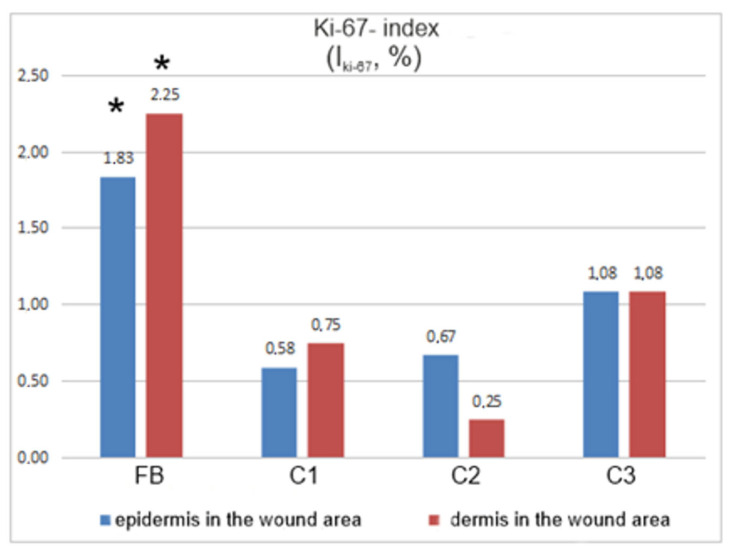
Index of Ki-67-positive nuclei (I_Ki-67_, %) in epidermis and dermis of regenerating burn wound 14 days after the end of treatment. FB—fibroblasts in a gel scaffold; C1—control group 1 (no treatment); C2—control group 2 (Chloramphenicol and methyluracil ointment (Laevomecolum)); C3—control group 3 (empty gel scaffold without fibroblasts). *—significant differences as compared to C1, C2, C3 values (*p* ≤ 0.05).

The proliferation of cells is a feature of regeneration. Therefore, cells specimens were stained with an AB agaist Ki-67, a protein marker of proliferating cells. The staining was quantified using a Ki-67 index (see Section 2). In animals treated with fibroblasts in gel, the proliferative index Ki-67 in the epidermis and dermis of the injury zone was 1.7–3.1 times statistically significantly (*p* ≤ 0.05) higher than in control groups 1–3 (Figure 7).

**Figure 8:**
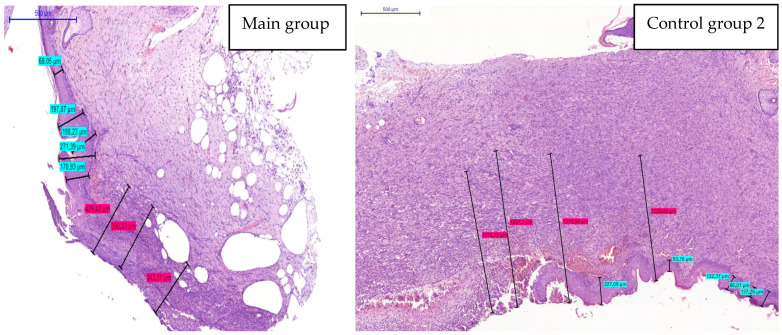
Epidermis thickness and depth of inflammatory infiltration in the wound edge section (μm) 14 days after the application of preparations to the surface of thermal burn. Hematoxylin and eosin staining. Turquise labels – epidermis, red labels – inflammatory infiltration. 400×. Scale bars are shown in the images.

Positive dynamics of burn wound healing in animals treated with a gel scaffold impregnated with fibroblasts were confirmed by morphometric assessment of epidermis thickness and depth of inflammatory infiltration in the regeneration period (Figure 8).

At day 14 after the end of the treatment with fibroblasts in a gel, the median epidermal thickness in the wound edge layer was 1.33 times greater (*p* ≤ 0.05) than in the control group 2 (Laevomecolum) animals, indicative of active primary epithelization. In the group treated with the fibroblasts in a gel, in the tissues adjacent to the regenerate zone, the number of granulocytes in the inflammatory infiltrate was 1.7 times less (*p* ≤ 0.05) than in the wound sections of animals treated with Laevomecolum. Total cell count was also increased significantly in the wounds of fibroblasts-treated animals as compared to the group treated with Laevomecolum (Table 3).

**Figure 9:**
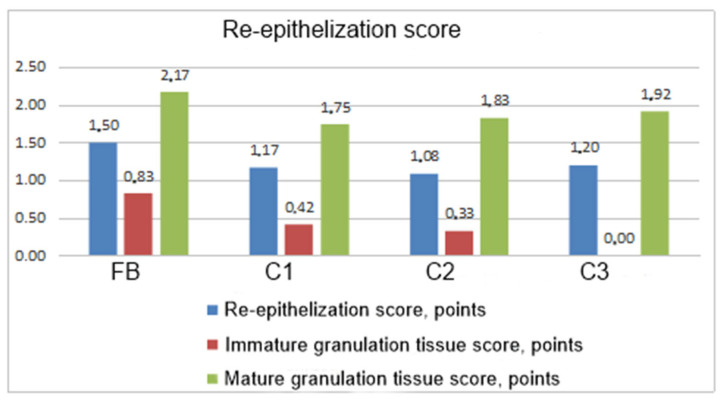
Comparative morphometric characterization of burn wound regeneration 14 days after application of dermal fibroblasts in hydroxyethylcellulose gel scaffold. FB—fibroblasts in a gel scaffold; C1—control group 1 (no treatment); C2—control group 2 (Chloramphenicol and methyluracil ointment (Laevomecolum)); C3—control group 3 (empty gel scaffold without fibroblasts).

In general, the intensity of burn wound regeneration assessed according to the re-epithelization score, immature granulation tissue, and mature granulations’ appearance indicated the advantages of the cell product compared to the control groups of animals (Figure 9).

### 3.7. Additional Study Outcomes

The additional study outcomes revealed that there were no significant differences in the wound-healing effect between Laevomecolum ointment (control group 2) and the control groups 1 (no treatment) and 3 (empty scaffold) during the regeneration phase. The result is likely attributable to the selected duration of ointment application (for five days), which resulted in a comparable period of retention of wound dressing in comparison with applications in other groups. In future studies, it is recommended that the duration of local treatment of experimental burn wounds with Laevomecolum ointment be extended, with a scheme close to the clinical one.

### 3.8. Adverse Events

No adverse events were observed during the study.

Summarizing the results of the study, in experiments on male rats, a single treatment of a deep dermal thermal burn with rat fibroblasts embedded in a HEC gel demonstrated enhanced reparative processes in the burn wound, formation of histotypic epithelio-synthetic tissue regenerate, and a decrease in the healing period compared to the effect of a standard reference preparation (Laevomekolum ointment). 

## 4. Discussion

The necessity for the development of efficacious wound-healing agents for the treatment of skin burns remains a significant and ongoing concern, as evidenced by the literature [30,31]. The primary objectives of the wound-healing product development are to restore the delayed re-epithelialization rate, facilitate the formation of a full-fledged dermal regenerate, and reduce the local inflammatory reaction caused by a posttraumatic increase in the level of proinflammatory cytokines (tumor necrosis factor alpha—TNFα; interleukins IL-1, IL-6, IL-12) [32,33]. At present, wound dressings that have a stimulatory effect on wound healing have been identified as a discrete category, with preparations based on cultured skin cells occupying a distinctive position within it [34]. Dermal fibroblasts remain the most promising tool for treating burn injuries [11,35,36]. The wound-healing effects of cultured fibroblasts are attributed to their capacity to synthesize extracellular matrix components and growth factors that facilitate the proliferation of autologous fibroblasts and keratinocytes. This process culminates in the formation of a complete dermal stroma and epidermalization [37]. Skin fibroblasts are a multifunctional heterogeneous group of cells that exhibit high morphological plasticity. They are involved both in inflammation and proliferation phases of the wound-healing process [38,39] During the inflammatory phase, activated fibroblasts engage a crosstalk that strengthens the local immune response via producing proinflammatory cytokines (TNF-α, IFN-γ, IL-6, IL-12, and releasing a wide range of C–C and C–X–C chemokines (e.g., CXCL1, CX3CL1, and CCL2) and juxtacrine interactions via ICAM1 and CD40 expression [39]. In proliferation stage, fibroblasts proliferate and contribute to angiogenesis and the formation of granulation tissue by secreting proangiogenic molecules, including vascular endothelial growth factor (VEGF), FGF, angiopoietin 1 (Ang-1), etc. At this stage, fibroblasts are able to transform into myofibroblasts—a conversion that underlies their fibrotic mechanism and wound contraction/closure ability. This healing-related differentiation of fibroblasts is triggered by mechanical signaling, cytokines, and growth factors, with transforming growth factor-beta (TGF-b) serving as a key mediator [38]. Myofibroblasts regulate wound contraction and tissue remodeling by combining the ability to synthesize ECM proteins and assume a contractile phenotype. Finally, cellularization (including myofibroblasts) decreases through apoptosis (or programmed cell death) [39]. Nevertheless, despite the impressive evidence of the promising clinical use of dermal equivalents in combustiology [3,11,12], there is no fibroblasts-based advanced technology medicinal product (ATMP) authorized for use in medical practice for burn treatment.

The main task of burn injury modeling is standardization. In this case, the depth of the lesion, on which the pathogenetic mechanisms of burn wound healing depend, is of paramount importance. Deep thermal burn followed by early (1 day) radical excision of the damaged skin down to the fascia (the fascia and underlying layers remain intact) has been modeled in rats. This animal model corresponds to the clinical guidelines, where active surgical tactics based on early excision of dead tissue in the area of burn with simultaneous or delayed skin grafting is recognized as the most rational [18,40]. Early excision leads to a rapid reduction in the area of burn wounds, reduces the potential for the development of infectious complications, and improves functional and cosmetic outcomes. The necrectomy was performed to create the most difficult conditions of wound healing, which, at the same time, are close to the clinical process of wound healing and therapy (see Supplementary File, the Section “Material and Methods”). The model makes it possible to standardize the possibilities of experimental treatment and evaluate the influence of wound dressings on the dynamics of healing through the intensity of marginal epithelialization, as well as the formation of granulation tissue with its subsequent regenerative remodeling. The rat subcutaneous muscle (panniculus carnosus) is involved in the contraction, although it is not a single contraction-promoting factor [41]. In our experimental model, wound edges were fixed with sutures to limit rodent-typical wound retraction in the postoperative period. Mechanical fixation of the wound edges prevents contraction and provides tension, making the wound-healing process closer to that of humans [41]. In small (up to 5 cm) wounds, contraction contributed 88 percent to wound closure, whereas the deposition of scar only contributed 12 percent [42]. In larger burns, the wound contraction did not work. However, wound contraction is different from “contracture”, which should be avoided during burn therapy. On day 11, myofibroblasts appeared in the wound, with 10% of fibroblasts [42]. Myofibroblasts and fibroblasts are two closely related cell types, and transitions from fibroblasts to myofibroblasts are well described [33]. Their main task in the process of wound healing in humans is the synthesis of collagen and regulation of inflammatory and regeneration processes. The presence of fibroblasts in the wound from this point of view is a positive point. Deep thermal burn with fascial necrectomy and mechanical fixation of the wound edges proceeded in rats by the type of incomplete reparative regeneration with delayed epidermalization and scar tissue formation. Application of Chloramphenicol and methyluracil ointment (Laevomecolum) limited destructive changes of epidermis and dermis in the inflammatory phase of the wound-healing process. In the regenerative period, the formation of granulation tissue, its maturation, and epithelization were delayed; in the central zone of the regeneration after the end of the treatment, incomplete epidermalization in combination with an inhibition of the process of connective tissue maturation with the preservation of areas of immature granulation tissue was observed. AMTP made of fibroblasts and hydroxyethylcellulose scaffold had an active wound-healing effect in the model of deep thermal burn in our study. Most likely, fibroblasts are the main contributors to marginal epithelialization (but also to the epithelialization in the wound’s center) because they secrete many growth factors that promote, among other things, proliferation and migration of keratinocytes. Myofibroblasts are indeed responsible for wound contraction [41], so the contribution of these cells to contraction may occur in our case. However, myofibroblasts are fewer in number compared to fibroblasts [42], so the main contribution of fibroblasts to healing is epithelialization. A single application of fibroblasts to the burn wound promoted a decrease in the signs of inflammation, accelerated the decrease of the burn area, and promoted complete healing of the lesion in the regenerative phase of the wound-healing process (Figure 5, Figure 6, Figure 7, Figure 8 and Figure 9) compared to the reference treatment that is a clinical standard. The results of the experiments illustrate the ability of the AMTP based on fibroblasts in gel scaffold to activate the processes of reparative regeneration in burn wounds according to all the criteria used in the study. 

The key factor in the treatment of burns using cell-based technologies is the early (within 10 days of trauma) application of cells to the surface of the defect [43]. In this context, the use of products based on expanded allogeneic cells is more feasible because they can be manufactured in advance. There are many examples of cell-based products based on cultured fibroblasts in clinical practice around the world, among which the best known are Dermagraft^®^ [44], Transcyte™ [45] (Advanced BioHealing, La Jolla, CA, USA), and Hyalograft 3D™ (Fidia Advanced Biopolymers, Abano Terme, Italy), which are polymeric two-dimensional coatings seeded with cells. The immunomodulatory effects of fibroblasts in the inflammatory period of wound healing can be affirmed; at present, skin grafts are mostly used to seal the wound by using their proliferation and differentiation abilities [46]. The development and introduction into clinical practice of a product based on expanded fibroblasts may become a quality alternative to currently used wound coatings due to the convenience of application and the lack of trauma to the wound surface when removing the wound dressing.

The results of the planimetric study indicate a positive dynamic of wound healing during the experiment in all groups included in the study. The absence of significant differences in the contraction rate of the control 3 group indicates the slow nature of wound healing due to the appearance of cocci infection, which is consistent with the literature [47]. 

The transition from necrotic type to inflammatory, bypassing degenerative-inflammatory-type cytograms in control group 2 on the 9th day of the study, confirms the results of the analysis of healing indices: the specific effect of the application of Laevomecolum, a standard treatment for burns treatment, was observed only in the course of treatment. Despite the fact that in control group 2, the change of the cytogram type from inflammatory to inflammatory-regenerative took place already on the 12th day of the experiment, this transition turned out to be unstable: on the 16th day, the group showed a return to the inflammatory profile by all the observed indices, which could be explained by the emergence of another cycle of local inflammation due to reinfection. In the main group, there was a transition from type degenerative-inflammatory to regenerative type cytograms, bypassing the inflammatory stage by 12 days of the experiment, but unlike control group 2, this transition was stable, which subsequently led to the regenerative profile of the cytograms of this group by 23 days. In the clinical practice of large deep burn treatment, the most widely accepted treatment is mesh auto-skin grafting. Generally, a 1.5- or 3-fold extended mesh auto-skin graft is used because it usually results in successful epithelization. In practice, a mesh auto-skin graft is applied on the debrided wound surface, on which a conventional ointment gauze dressing is placed to protect the mesh auto-skin graft. Kashiwa et al. evaluated a biologic based on fibroblasts and hyaluronic acid and atelo-collagen dressing for highly expanded mesh autologous skin grafts. When applied to the six-fold-expanded autologous skin graft, it produced growth factors and extracellular matrix components that promote tissued granulation and epithelialization of the skin. Moravvej et al. [48] cultured allogeneic fibroblasts on a combination of silicone, glycosaminoglycan, and autologous mesh grafts. Both groups demonstrated the significant increase in rate of epithelization. We demonstrated that fibroblasts embedded in biologically neutral very inexpensive gel preserve their ability to promote the regeneration of skin.

## 5. Conclusions

In the present study, we proved the specific activity of dermal fibroblasts in HEC gel in animal model. These findings provide a rationale for further preclinical studies with the objective of developing ATMP.

### Limitations of the Study

Factors limiting the conclusions of the study may be due to the conditions of the experiments, species and sex differences of the experimental animals, the scope of statistical sampling, and the methodological approaches used, which are considered by the program of preclinical studies and will be addressed. Another limitation of the study is the difference in the burn wound-healing process between rodents and humans. In rodents, wound contraction is greater. The area of defect is smaller in absolute value than a large burn in humans. These factors result in different rate of wound closure and epithelization. In our study, we minimized the wound contraction factor by mechanical fixation of the wound edges and demonstrated the role of fibroblasts in regeneration. However, the differences between animal models and humans should be considered.

## Figures and Tables

**Table 1:** Number of animals in the experimental groups.

Treatment	Tests	Complex I	Complex II
No treatment (Control 1)	**Complex I** Visual assessment of the wound surface; planimetry of wounds; cytologic wound prints **Complex II** Qualitative and quantitative histomorphology; immunohistochemistry	13	12
Chloramphenicol and methyluracil ointment (Laevomecolum) (Control 2)		13	12
Hydroxyethylcellulose gel without fibroblasts (Control 3)		13	12
Fibroblasts embedded in hydroxyethylcellulose gel (Main group)		13	12
		52	48
Total:		100	

**Table 2:** Area of burn wounds (cm^2^) at different time points following the application of preparations to the surface of the thermal burn ♦.

Animal Groups		Before Treatment (96 h after Burn)	Duration of Observation Period, Days				
			1	3	7	14	21
No treatment (Control 1)	Me	13.45	12.35	9.63	7.12	4.54	2.57
	Q1; Q3	12.93; 13.86	11.41; 12.86	9.10; 9.94	6.27; 7.74	4.31; 5.41	2.26; 3.12
	n	25	23	21	19	17	15
Chloramphenicol and methyluracil ointment (Laevomecolum) (Control 2)	Me	13.57	8.97 *	6.17 *	4.55 *	2.33 *	1.28 *
	Q1; Q3	13.15; 14.26	8.45; 9.78	5.40; 6.89	3.94; 5.01	1.66; 2.72	0.86; 2.02
	n	25	23	21	19	17	15
Hydroxyethylcellulose gel without fibroblasts (Control 3)	Me	13.22	11.42 **	8.87 **	6.37 **	3.81 *^,^**	1.61 *
	Q1; Q3	12.47; 13.73	10.39; 12.34	8.40; 9.42	5.82; 7.20	2.85; 4.33	1.24; 2.25
	n	25	23	21	19	17	15
Fibroblasts embedded in hydroxyethylcellulose gel (Main group)	Me	13.81	10.44 **	6.57 *^,^***	3.94 *^,^**^,^***	1.42 *^,^**^,^***	0.25 *^,^ **^,^***
	Q1; Q3	13.36; 14.29	10.03; 11.66	6.10; 7.18	3.55; 4.33	1.19; 1.69	0.18; 0.47
	n	25	23	21	19	17	15
*p* (Yes)		≤0.05	≤0.05	≤0.05	≤0.05	≤0.05	≤0.05

♦ n—number of animals; Me—median; Q1; Q3—lower and upper quartiles; *p* (Yes)—significance of the Kruskell–Wallis rank analysis of variance; * significant differences (*p* ≤ 0.05) compared to control 1; ** significant differences (*p* ≤ 0.05) compared to control 2; *** significant differences (*p* ≤ 0.05) compared to Control 3.

**Table 3:** Histomorphometric examination of the burn wound area 14 days after the end of treatment.

Morphometric Parameters	Main Group (Treatment with Fibroblasts Embedded in a Gel Scaffold) n = 3	Control Group 2 n = 3
Epidermis thickness, μm	147.7 [85.2; 248.2] *	85.4 [85.3; 152.7]
Granulocytes per field of view	11 [9; 29] *	25 [14; 45]
Total cell count in the wound area per field of view	261 [145; 325] *	164 [156; 187]

Data are shown as Me [Q1; Q3]—median and [Q1, Q3 quartile], n—number of animals, * differences are significant (*p* ≤ 0.05) compared to control group 2 (treatment with Chloramphenicol and methyluracil ointment (Laevomecolum).

## Data Availability

Data are contained within the article and Supplementary Materials.

## Supplementary Materials

The following supporting information can be downloaded at: https://www.mdpi.com/article/10.3390/biomedicines12102215/s1, Supplentary File containing: (1) Supplementary Figure S1 (Burn position (A) and depth (B)). (2) Supplementary Figure S2 (Viscosity (cP) versus velocity (reverse per minute, RPM) plot demonstrating a non-Newtonian pseudo-plastic flow behaviour of both the emty HEC gel and the gel after mixing with fibroblasts resuspended in 0.9% sodium saline) and (2) Supplementary Table S1 (Viscosity (cps) versus velocity (rpm) measurements).

## References

[1] Social Determinants of Health (SDH), World Health Organization (WHO) (2008). A WHO Plan for Burn Prevention and Care.

[2] Legrand M., Barraud D., Constant I., Devauchelle P., Donat N., Fontaine M., Goffinet L., Hoffmann C., Jeanne M., Jonqueres J. (2020). Management of Severe Thermal Burns in the Acute Phase in Adults and Children. Anaesth. Crit. Care Pain Med..

[3] Domaszewska-Szostek A.P., Krzyżanowska M.O., Czarnecka A.M., Siemionow M. (2021). Local Treatment of Burns with Cell-Based Therapies Tested in Clinical Studies. J. Clin. Med..

[4] Bozo I.I., Deev R.V., Pinaev G.P. (2010). Is “Fibroblast” a Specialized Cell or a Functional Condition of Mesenchymal Cells Derivatives?. Tsitologiia.

[5] Horch R.E., Wagner G., Bannasch H., Kengelbach-Weigand A., Arkudas A., Schmitz M. (2019). Keratinocyte Monolayers on Hyaluronic Acid Membranes as “Upside-Down” Grafts Reconstitute Full-Thickness Wounds. Med. Sci. Monit..

[6] Silva A.C., Oliveira M.R., Amaral L.F.A., Ferreira S., Garcia I.R., Mariano R.C. (2016). Effect of Doxycycline in Gel Form on Bone Regeneration: Histomorphometric and Tomographic Study in Rat Calvaria. J. Periodontol..

[7] SMYTH H.E., CARPENTER C.P., WEIL C.S. (1947). The Chronic Toxicity of Hydroxyethyl Cellulose for Rats. J. Am. Pharm. Assoc. Am. Pharm. Assoc..

[8] Noreen A., Zia K.M., Tabasum S., Khalid S., Shareef R. (2020). A Review on Grafting of Hydroxyethylcellulose for Versatile Applications. Int. J. Biol. Macromol..

[9] El Fawal G.F., Abu-Serie M.M., Hassan M.A., Elnouby M.S. (2018). Hydroxyethyl Cellulose Hydrogel for Wound Dressing: Fabrication, Characterization and in Vitro Evaluation. Int. J. Biol. Macromol..

[10] Zulkifli F.H., Hussain F.S.J., Rasad M.S.B.A., Mohd Yusoff M. (2014). Nanostructured Materials from Hydroxyethyl Cellulose for Skin Tissue Engineering. Carbohydr. Polym..

[11] Vagner D.O., Zinoviev E.V., Krylov K.M., Krylov P.K., Soloshenko V.V., Kostyakov D.V., Yurkevich Y.V., Enukashvily N.I., Blinova M.I., Aleksandrova O.I. (2018). Experience in the Clinical Use of Allogeneic Fibroblasts in Patients with Severe Burns. Her. North-Western State Med. Univ. Named After II Mechnikov.

[12] Sierra-Sánchez Á., Kim K.H., Blasco-Morente G., Arias-Santiago S. (2021). Cellular Human Tissue-Engineered Skin Substitutes Investigated for Deep and Difficult to Heal Injuries. NPJ Regen. Med..

[13] Bakker O.J., van Santvoort H.C., van Brunschot S., Geskus R.B., Besselink M.G., Bollen T.L., van Eijck C.H., Fockens P., Hazebroek E.J., Nijmeijer R.M. (2012). Endoscopic Transgastric vs Surgical Necrosectomy for Infected Necrotizing Pancreatitis. JAMA.

[14] (2014). Guidelines for Accommodation and Care of Animals. Environment, Housing and Management.

[15] Tikhomirova A.V., Goryachev D.V., Merkulov V.A., Lysikova I.V., Gubenko A.I., Zebrev A.I., Solovieva A.P., Romodanovsky D.P., Melnikova E.V. (2018). Preclinical and Clinical Aspects of the Development of Biomedical Cell Products. Bull. Sci. Cent. Expert Eval. Med. Prod..

[16] Gouma E., Simos Y., Verginadis I., Lykoudis E., Evangelou A., Karkabounas S. (2012). A Simple Procedure for Estimation of Total Body Surface Area and Determination of a New Value of Meeh’s Constant in Rats. Lab. Anim..

[17] Mosier M.J., Gibran N.S. (2009). Surgical Excision of the Burn Wound. Clin. Plast. Surg..

[18] Zinoviev E.V., Vagner D.O., Chukharev A.E. (2023). A New Method for Determining the Volume of Blood Loss during Necrectomy in Patients with Deep Burns. Burn Care Prev..

[19] Dovnar R.I. (2020). Nuances of the Choice of Experimental Animals for Modeling the Healing Process of the Skin Wound. J. Grodno State Med. Univ..

[20] Bhuyan C., Saha D., Rabha B. (2021). A Brief Review on Topical Gels as Drug Delivery System. J. Pharm. Res. Int..

[21] Boutelier D., Cruden A., Saumur B. (2016). Density and Visco-Elasticity of Natrosol 250 HH Solutions: Determining Their Suitability for Experimental Tectonics. J. Struct. Geol..

[22] Enukashvily N.I., Kotkas I.E., Bogolyubov D.S., Kotova A.V., Bogolyubova I.O., Bagaeva V.V., Levchuk K.A., Maslennikova I.I., Ivolgin D.A., Artamonov A.Y. (2020). Detection of Cells Containing Internalized Multidomain Magnetic Iron (II, III) Oxide Nanoparticles Using the Magnetic Resonance Imaging Method. Tech. Phys..

[23] Liebert M.A. (1986). Final Report on the Safety Assessment of Hydroxyethylcellulose, H Yd Roxy pro Py Ice1 I u Lose, Met h Ylcellu Lose, H Yd Roxy pro Pyl Methylcellulose, and Cellulose Gum. J. Am. Coll. Toxicol..

[24] Samojlova A.V., Gostyukhina A.A., Bol’shakov M.A., Rostov V.V. (2022). Effect of Stimulation of Healing of Burn Wounds in Rats with Nanosecond Microwave Pulses. Mod. Iissues Biomed..

[25] Fenchin K.M. (1979). Wound Healing.

[26] Ioffe O.Y., Stetsenko O.P., Kindzer S.L., Kryvopustov M.S., Tsiura Y.P., Prykhodko Y.S. (2023). Application of Probiotic Antisepsis for Purulent Complications in Patients with Type 2 Diabetes Mellitus. Wiad. Lek..

[27] Domische M.Y., Maliar A.V., Maliar V.V., Maliar V.V., Maliar V.A. (2022). Monitoring Assessment of the Early Process on the Background of Tes Therapy. Wiad. Lek..

[28] Sergel O.S., Goncharova Z.G., Teplyakov V.G., Kuzin M.I., Kosyuchenok B.M. (1990). Cytological Studies. Wound and Wound Infection.

[29] An J., Beauchemin N., Albanese J., Abney T.O., Sullivan A.K. (1997). Use of a Rat CDNA Probe Specific for the Y Chromosome to Detect Male-Derived Cells. J. Androl..

[30] Wong T., McGrath J.A., Navsaria H. (2007). The Role of Fibroblasts in Tissue Engineering and Regeneration. Br. J. Dermatol..

[31] Su L., Jia Y., Fu L., Guo K., Xie S. (2023). The Emerging Progress on Wound Dressings and Their Application in Clinic Wound Management. Heliyon.

[32] Singh S., Young A., McNaught C.-E. (2017). The Physiology of Wound Healing. Surgery.

[33] Bainbridge P. (2013). Wound Healing and the Role of Fibroblasts. J. Wound Care.

[34] Heras K.L., Igartua M., Santos-Vizcaino E., Hernandez R.M. (2022). Cell-Based Dressings: A Journey through Chronic Wound Management. Biomater. Adv..

[35] Melnikova E.V., Merkulova O.V., Borisevich I.V., Merkulov V.A. (2018). From Cellular Technologies to Biomedical Cell Products: Practice in the Use of Drugs Based on Viable Human Cells in the Russian Federation. Tsitologiya.

[36] Li Z., Maitz P. (2018). Cell Therapy for Severe Burn Wound Healing. Burn. Trauma.

[37] Tracy L.E., Minasian R.A., Caterson E.J. (2016). Extracellular Matrix and Dermal Fibroblast Function in the Healing Wound. Adv. Wound Care.

[38] Knoedler S., Broichhausen S., Guo R., Dai R., Knoedler L., Kauke-Navarro M., Diatta F., Pomahac B., Machens H.G., Jiang D. (2023). Fibroblasts—The Cellular Choreographers of Wound Healing. Front. Immunol..

[39] Cialdai F., Risaliti C., Monici M. (2022). Role of Fibroblasts in Wound Healing and Tissue Remodeling on Earth and in Space. Front. Bioeng. Biotechnol..

[40] Soloshenko V., Wagner D.O., Kostyakov D.V., Kourov A.S., Chukharev A.E., Gogokhiya T.Z., Movchan K.N., Derii E.K., Zinoviev E.V. (2023). Possibilities of Reducing Blood Loss during Tangential Necrectomy in Burned Patients (Literature Review). Russ. Biomed. Res..

[41] Son D.O., Hinz B. (2021). A Rodent Model of Hypertrophic Scarring: Splinting of Rat Wounds. Methods Mol. Biol..

[42] Berry D.P., Harding K.G., Stanton M.R., Jasani B., Ehrlich H.P. (1998). Human Wound Contraction: Collagen Organization, Fibroblasts, and Myofibroblasts. Plast. Reconstr. Surg..

[43] Klama-Baryła A., Kitala D., Łabuś W., Kraut M., Glik J., Nowak M., Kawecki M. (2018). Autologous and Allogeneic Skin Cell Grafts in the Treatment of Severely Burned Patients: Retrospective Clinical Study. Transplant. Proc..

[44] Marston W.A., Hanft J., Norwood P., Pollak R., Dermagraft Diabetic Foot Ulcer Study Group (2003). The Efficacy and Safety of Dermagraft in Improving the Healing of Chronic Diabetic Foot Ulcers: Results of a Prospective Randomized Trial. Diabetes Care.

[45] Kumar R.J., Kimble R.M., Boots R., Pegg S.P. (2004). Treatment of Partial-thickness Burns: A Prospective, Randomized Trial Using Transcyte TM. ANZ J. Surg..

[46] Wang Z., Qi F., Luo H., Xu G., Wang D. (2022). Inflammatory Microenvironment of Skin Wounds. Front. Immunol..

[47] Marcato P.D., De Paula L.B., Melo P.S., Ferreira I.R., Almeida A.B.A., Torsoni A.S., Alves O.L. (2015). In Vivo Evaluation of Complex Biogenic Silver Nanoparticle and Enoxaparin in Wound Healing. J. Nanomater..

[48] Moravvej H., Hormozi A.K., Hosseini S.N., Sorouri R., Mozafari N., Ghazisaidi M.R., Rad M.M., Moghimi M.H., Sadeghi S.M., Mirzadeh H. (2016). Comparison of the Application of Allogeneic Fibroblast and Autologous Mesh Grafting With the Conventional Method in the Treatment of Third-Degree Burns. J. Burn Care Res..

