# Supplementary material for: The Wound-Healing Effect of a Novel Fibroblasts-Impregnated Hydroxyethylcellulose Gel in a Rat Full-Thickness Burn Model: A Preclinical Study
Source: Biomedicines. 2024 Sep 28;12(10):2215. doi: 10.3390/biomedicines12102215 (PMC11505042; doi:10.3390/biomedicines12102215)
Supplement: Supplementary file 1 [file biomedicines-12-02215-s001.zip › biomedicines-3142336-supplementary.pdf]

biomedicines

MDPI

Article

The wound-healing effect of fibroblasts in the rat dermal burn model: a preclinical study.

Yury A. Novosad[1,2], Anton S. Shabunin[1], Natella I. Enukashvily[3, 4,5], Olga V. Supilnikova[3,4], Anastasia I. Konkina[3] Natalia Yu. Semenova[6], Gleb S. Yatsemirsky[3], Evgenii V. Zinoviev[7], Kristina N. Rodionova[1,2], Kirill L. Kryshen[8], Antonina Yu. Borodina[8], Alexander Yu. Makarov[1], Andrey.M. Fedyuk[1], Alexander D. Nilov[1], Elena V. Chikulaeva[1], Lidiya S. Konkova[1], Irina S. Chustrak[1], Veronika V. Traxova[1], Platon A. Safonov[1], Sergey V. Vissarionov[9], Egor M. Prikhodko[3,10*], Yury.V. Yurkevich[3*]

[1] Professor G.I. Gaivoronsky laboratory of experimental traumatology and orthopedics with vivarium, H. Turner National Medical Research Center for Children's Orthopedics and Trauma Surgery (196603, St. Petersburg, Russia)

[2] Institute of Biomedical Systems and Biotechnologies, Peter the Great St.Petersburg Polytechnic University (195251 St. Petersburg, Russia);

[3] Cell Technology Center Pokrovsky, 199066 St. Petersburg, Russia),

[4]. Cell Technologies Lab., North-Western State Medical University named after I.I. Mechnikov, 191015 St. Peterburg, Russia.

[5]. Lab of the non-coding DNA Study., Institute of Cytology, 194064, St. Peterburg, Russia.

[6] Research Department of Pathomorphology, Center for Preclinical and Translational Research, Federal State Budgetary Institution « Almazov National Medical Research Centre» of the Ministry of Health of Russia; 199034, St. Petersburg, Russia.

[7]. Saint-Petersburg I. I. Dzhanelidze Research Institute of Emergency Medicine, 192242 St. Petersburg, Russia, evz@list.ru (E.V.Z.)

[8]. "Home of Pharmacy" Center, Leningrad region 188663, Russian Federation

[9] H. Turner National Medical Research Center for Children's Orthopedics and Trauma Surgery (196603, St. Petersburg, Russia)

[10] Institute of Medicine, St. Petersburg State University, 199034, St. Petersburg, Russia

* Correspondence: yurkevich2@yandex.ru (Yu.V.Yu.), ceo@pokrovcell.ru (E.M.P.), vissarionovs@gmail.com (S.V.V.)

Supplementary information

Materials and methods

Additional details of medical interventions

The depth of the skin burn damage and its localisation on the animal body are shown in Supplementary Figure S1).

A B

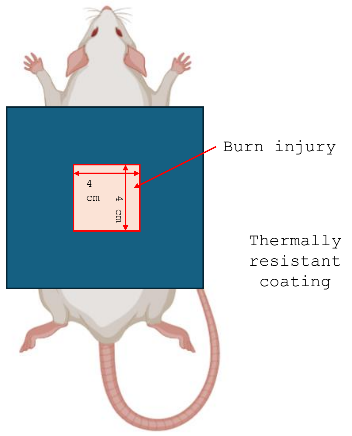

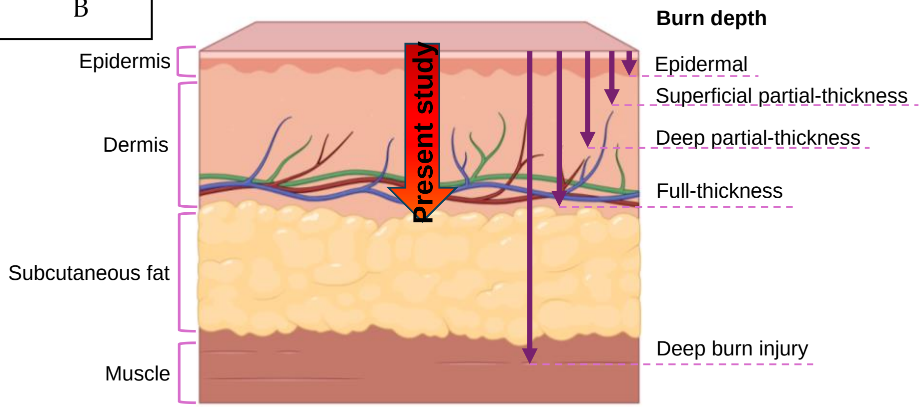

Supplementary Figure S1. Burn position (A) and depth (B).

Twenty-four hours after the burn, the lesion was excised as described in the main text. This procedure, termed radical necrectomy, aligns with the clinical practice of burn treatment and includes the removal of damaged tissues in the lesion area down to the fascia.Necrectomy is recommended for full-thickness burns and mixed partial-thickness+full-thickness burns according to national clinical guidelines. A burn scab consists of denatured proteins and necrotized skin components. As the local inflammatory response develops, autolytic rejection begins beneath the scab and is enhanced by the inflammatory response. Because a burn scab can be a source of infection leading to septic complications, it is important to remove the scab as soon as it forms. The process of removing non-viable, damaged and infected tissue and tissue debris from the wound surface to enhance the healing of potentially healthy tissue is commonly referred to as debridement. In current medical practice, surgical necrectomy (as well as chemical and enzymatic approaches) is most commonly used to remove eschar. In laboratory practice, necrectomy in combination with wound edges fixation decreases the wound contraction, that is typical for rodents.

Gel viscosity evaluation

A Brookfield rotational viscometer (USA) was used to measure dynamic viscosity at room temperature and at 37°C. Spindle #6 was selected for the measurements. The measurements were performed at rotation speeds from 0.3 to 100 rpm.

Results

The viscosity of HEC gel without cells as well as the viscosity of the same gel after addition of fibroblasts resuspended in the gel as described in the article body text was measured (Supplementary Figure S2)

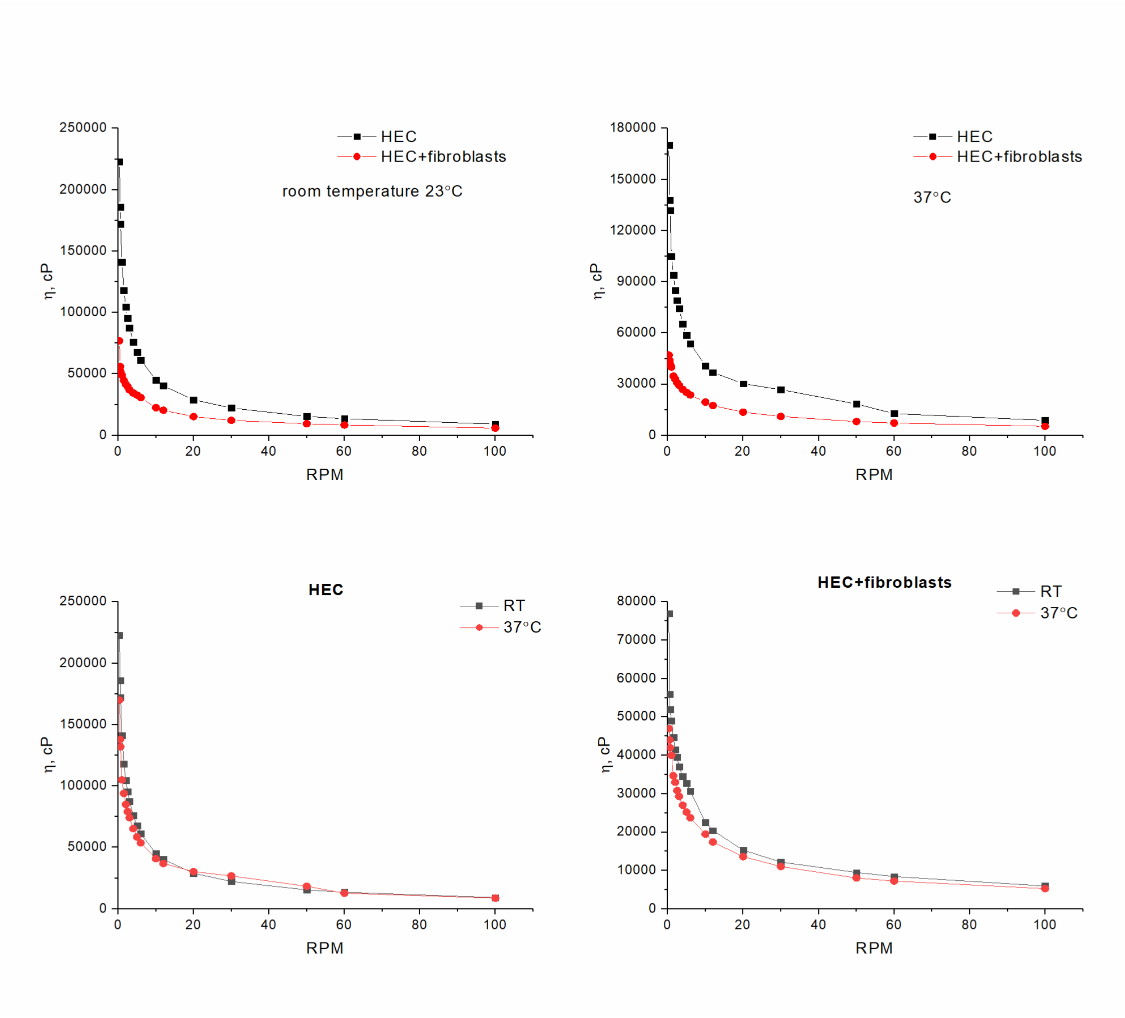

Supplementary Figure S2. Viscosity (cP) versus velocity (reverse per minute, RPM) plot demon-strating a non-Newtonian pseudo-plastic flow behaviour of both the emty HEC gel and the gel after mixing with fibroblasts resuspended in 0.9% sodium saline. *X-axys* – velocity (RPM), *Y-axys* – viscosity (centipoise, cP)

Supplementary Table S1. Viscosity (cps) versus velocity (rpm) measurements

	**Viscosity (cP)**			
	HEC		HEC+fibroblasts	
Velocity, RPM	RT	37°C	RT	37°C
0,3	223000	170000	77000	47000
0,5	186000	138000	56000	44000
0,6	172000	132000	52000	42000
1	141000	105000	49000	40000
1,5	118000	94000	44700	34700
2	104500	85000	41500	33000
2,5	95600	79200	39600	30800
3	87700	74300	37000	29300
4	76000	65300	34500	27000

5	67800	58600	32800	25200
6	61300	53700	30700	23700
10	45000	40800	22500	19500
12	40330	36920	20420	17420
20	29000	30330	15300	13600
30	22300	26800	12200	11030
50	15440	18400	9480	8040
60	13500	12730	8420	7250
100	9220	8800	5960	5280
